# Do major host shifts spark diversification in butterflies?

**DOI:** 10.1002/ece3.6116

**Published:** 2020-02-26

**Authors:** Chloe Kaczvinsky, Nate B. Hardy

**Affiliations:** ^1^ Department of Entomology and Plant Pathology Auburn University Auburn AL USA

**Keywords:** coevolution, evolutionary ecology, plant‐insect interactions, species diversification

## Abstract

The Escape and Radiate Hypothesis posits that herbivorous insects and their host plants diversify through antagonistic coevolutionary adaptive radiation. For more than 50 years, it has inspired predictions about herbivorous insect macro‐evolution, but only recently have the resources begun to fall into place for rigorous testing of those predictions. Here, with comparative phylogenetic analyses of nymphalid butterflies, we test two of these predictions: that major host switches tend to increase species diversification and that such increases will be proportional to the scope of ecological opportunity afforded by a particular novel host association. We find that by and large the effect of major host‐use changes on butterfly diversity is the opposite of what was predicted; although it appears that the evolution of a few novel host associations can cause short‐term bursts of speciation, in general, major changes in host use tend to be linked to significant long‐term decreases in butterfly species richness.

## Graphical abstract

We test several key predictions of the Escape and Radiate Hypothesis using comparative phylogenetics in nymphalid butterflies. We find that although it appears that the evolution of a few novel host associations may have caused bursts of speciation, in general, major changes in host use tend to be linked to significant decreases in butterfly species richness.

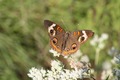

## 1 INTRODUCTION

About half of the 1.6 million described species are insects, and about half of all insect species are herbivores (Roskov et al., 2018). How did herbivorous insects come to be so diverse? The Escape and Radiate Hypothesis (Ehrlich & Raven, 1964; Thompson, 1989) posits a coevolutionary version of adaptive radiation. When a plant lineage evolves a new chemical defense, it escapes from its herbivores, enters a new adaptive zone, and diversifies ecologically and taxonomically. Reciprocally, when an herbivorous insect lineage evolves a counter‐adaptation to a plant chemical defense, it escapes from its competitors and diversifies. The Escape and Radiate Hypothesis can be traced back to Ehrlich and Raven's (1964) essay on coevolution, in which they surmise that “the fantastic diversification of modern insects had developed in large measure as the result of a stepwise pattern of coevolutionary stages.” To be clear, Ehrlich and Raven (1964) had coevolution per se as their main focus, and their comments on species diversification dynamics were left general and speculative. The name Escape and Radiate was introduced by Thompson (1989). Since then, several authors have sought to more fully develop the Escape and Radiate Hypothesis by making more specific and testable predictions about how host‐plant coevolution might affect the process and pattern of herbivorous insect species diversification (e.g., Althoff, Segraves, & Johnson, 2014; Fordyce, 2010; Hembry, Yoder, & Goodman, 2014; Janz, 2011; Janz & Nylin, 2008; Suchan and Alvarez, 2015). Here, we use the name Escape and Radiate as short‐hand for this extended set of predictions, several of which have yet to be tested (Futuyma & Agrawal, 2009).

Our current understanding of the Escape and Radiate Hypothesis is an extension of the basic theory of adaptive radiation (Schluter, 2000) and inherits many of the same underlying assumptions. To wit, it assumes that release from constraints on diversity will cause the speciation of specialists, rather than the niche expansion of generalists (Yoder et al., 2010). Hence, it predicts that the colonization of novel host groups will increase herbivorous insect species diversity. Implicit here is that both plant defenses and herbivorous insect diets are phylogenetically conservative (Futuyma & Mitter, 1996; Kergoat, Silvain, Delobel, Tuda, & Anton, 2007). Otherwise, the notion of coevolutionary adaptive zones becomes problematic; for example, evolving to overcome one plant species' defenses would not allow an insect population to overcome the defenses of a related plant species. Another assumption that the Escape and Radiate Hypothesis inherits from the general theory of adaptive radiation is that adaptive zones can be saturated. Hence, it predicts that the colonization of a novel host group will cause an immediate uptick and then a subsequent slowing of speciation rates as the novel adaptive zone is filled (Losos & Mahler, 2010). By extension, the Escape and Radiate Hypothesis predicts that some novel host associations should represent greater ecological opportunities and more expansive adaptive zones than others and that the dimensions of these zones should determine their effects on species diversity (Farrell & Mitter, 1994; Schluter, 2000).

### 1.1 Evidence for and against the Escape and Radiate Hypothesis

Evidence for the Escape and Radiate Hypothesis is mixed. Researchers have documented several putative cases of a plant lineage escaping from its herbivores and undergoing a subsequent burst of species diversification (Farrell, Dussourd, & Mitter, 1991; Futuyma & Agrawal, 2009). And for herbivorous insects, several phylogenetic studies have shown apparent links between particular host‐use shifts and upticks in diversity (Braby & Trueman, 2006; Futuyma & Agrawal, 2009; Wheat et al., 2007). Clear evidence of phylogenetic conservatism has been found for some plant defensive chemistries (e.g., Liscombe, Macleod, Loukanina, Nandi, & Facchini, 2005; Wink & Mohamed, 2003), but not others (e.g. Wink, 2003); currently, we lack a quantitative sense for the overall phylogenetic conservation of plant chemical defenses (Agrawal, 2007). Likewise, the phylogenetic conservatism of host use varies across clades of herbivorous insects (e.g., Janz & Nylin, 2008; Hardy, Gullan, & Hodgson, 2008). The Escape and Radiate Hypothesis would seem most applicable to groups such as butterflies for which the assumptions of phylogenetic conservation of host use and defensive chemistry in at least some host groups are met. To date, Fordyce's (2010) study of butterflies has been the most comprehensive test of macro‐evolutionary predictions of the Escape and Radiate Hypothesis. In it, he presents evidence for temporary increases in speciation rates after the evolution of a handful of major novel host associations, classified as such a priori. To be sure, such bursts of speciation are as expected under the Escape and Radiation Hypothesis (Fordyce, 2010), but, as it stands, we do not know if such effects are typical or exceptional; the prediction that major host shifts will spur diversification has yet to be tested with statistical rigor.

Here, we use phylogeny‐based statistical analyses of butterflies to address three key questions: Do major new host associations tend to cause (a) a burst of speciation (Fordyce, 2010) and (b) lasting increases in species diversity (Janz & Nylin, 2008)? And (c) do novel host groups affording greater ecological opportunity cause greater increases in butterfly diversity (Losos & Mahler, 2010; Schluter, 2000)?

## 2 METHODS

We use brush‐footed butterflies (Papilionoidea: Nymphalidae) as a model, since both their larval host associations and phylogenetic relationships are relatively well known. Although more is known of host use in some other groups of insects, for example scale insects (García Morales et al. 2016), in those groups less is known about phylogeny. We worked at two phylogenetic levels in nymphalids. First, we analyzed nymphalid genera, of which 398 are extant. We then analyzed a partial species‐level data set, covering 2,423 of the 6,431 extant species recognized in the Catalogue of Life database (Roskov et al., 2018). The genus‐level data for this study came from two main sources. Host‐use and species diversity data for nymphalid genera are from Hamm and Fordyce (2015), and phylogenetic data are from Wahlberg (2006). For species‐level analyses, host‐use data are from the lepidopteran HOSTS database (Robinson, Ackery, Kitching, Beccaloni, & Hernández, 2010), and phylogenetic data are from Peterson, Hardy, and Normark (2016). Below, we first describe the genus‐level analyses in detail and then explain how the species‐level analyses differed. To test the macro‐evolutionary predictions of the Escape and Radiate Hypothesis, we needed (a) reconstructions of ancestral host use of nymphalids, (b) quantifications of the scope of ecological opportunities opened by evolving specific novel host associations (host gains, for short), and (c) a statistical approach to evaluate how major host‐use changes affect speciation rates and extant species diversity.

### 2.1 Genus‐level analyses

#### 2.1.1 Reconstructions of ancestral host use

All analyses were performed in R (R Core Team, 2019). First, we used Dispersal Extinction Cladogenesis (DEC) models to reconstruct the phylogenetic history of the use of host orders and families (as in Hardy, 2017). Although DEC models were initially developed to estimate ancestral geographic ranges, they are well‐suited for the estimation of ancestral states for any multi‐state discrete character such as host use (Hardy, 2017). An alternative approach would have been to code the use or nonuse of each nymphalid host‐plant taxon as a binary trait and then use standard discrete trait models to independently reconstruct phylogenetic histories of the use of each host. However, simulations have shown that such an approach reconstructs ancestral host use with a strong bias toward the present and tends to infer ancestors without any hosts at all—a problem that DEC estimation avoids (Hardy, 2017). In our DEC models, the host use of each extant nymphalid genus and each ancestral nymphalid node is expressed as a combination of discrete host taxa. The feasibility of DEC modeling is limited by the size of the matrix which specifies the probabilities (or rates) of each type of possible host‐use transition. If this rate matrix is too large, computations are intractable. A full matrix, with terms for every possible combination of host‐plant taxa and for every possible change between those combinations, would have been unfeasible.

We took two approaches to keep rate matrices under 1,600 states (which kept analysis run times under 2 weeks). First, we reconstructed the history of associations between nymphalids and their twelve most commonly used host‐plant orders with a maximum nymphalid genus host‐breadth size of five orders. This required dropping 23 of the most polyphagous nymphalid genera, along with nine additional genera for which we lacked host‐use data, leaving 357 genera (89.6% of the total) for analysis. Second, we reconstructed ancestral use of host‐plant families over a set of nymphalid subclades using an R script (Appendix S1) to cut the phylogeny into the most inclusive set of nonoverlapping clades that comprised at least ten extant nymphalid genera and would result in DEC rate matrices with <1,600 states. We also excluded from consideration any host family used by only one nymphalid genus. This yielded nine nymphalid subtrees, encompassing 238 of the 398 genera and 51 host‐plant families. Note that this approach was not entirely inclusive and was biased against clades containing genera with especially broad host associations. The order‐level reconstructions were not subject to these biases, but host‐use variation at the level of families may be more biologically meaningful. Ehrlich and Raven (1964) suggested that many family‐level plant taxa can be traced back to defensive innovation, and many subsequent authors have used plant family diversity as a proxy for the diversity of their defensive chemicals (e.g., Fordyce, 2010; Hardy & Otto, 2014). Hence, we conducted analyses at both levels of host‐plant taxonomy.

**Figure 1:**
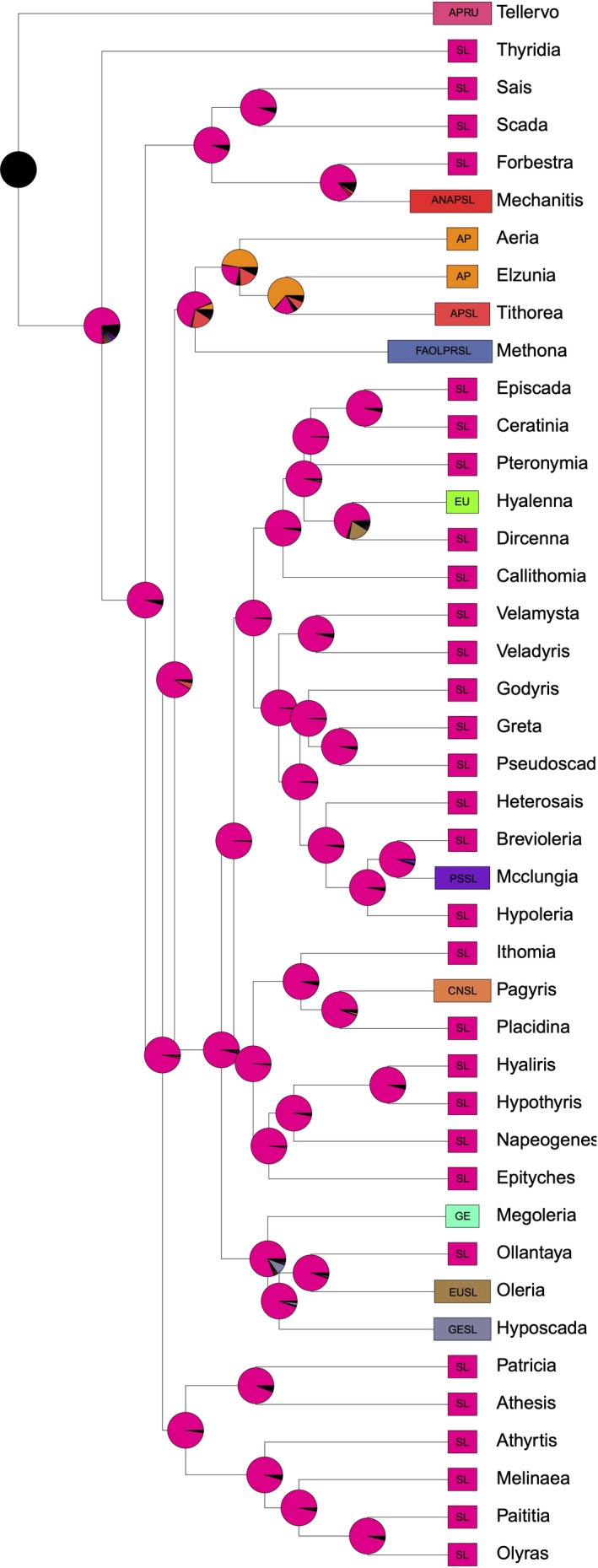
Example DEC* reconstruction of the use of host‐plant families over part of the nymphalid phylogeny. Each host‐use state has a unique color and the internal pie charts show the proportional likelihood of ancestral host‐use states. Host abbreviations: AP, Apocynaceae; RU, Rubiaceae; SL, Solanaceae; AN, Annonaceae; FA, Fabaceae; OL, Oleaceae; PR, Primulaceae; EU, Euphorbiaceae; PS, Passifloraceae; CN, Convolvulaceae; GE, Gesneriaceae

DEC estimations of ancestral host use were performed with the R package BioGeoBEARS (Matzke, 2013). Specifically, we used the DEC* model, which excludes the ancestral null state (Massana, Beaulieu, Matzke, & O'Meara, 2015) and thereby requires all ancestors to have a host, a constraint that we think reflects the biological reality (Massana et al., 2015; Matzke, 2014; See Figure 1 for an example of host‐use reconstruction; figures for all reconstructions are provided in Appendices [Link], [Link], [Link], [Link], [Link], [Link], [Link], [Link], [Link]).

#### 2.1.2 Linear model parameterization one—effects of host‐use changes on diversity

To test the predictions of the Escape and Radiate Hypothesis, we used a linear modeling approach. We first sought to explain the variation across nymphalid lineages in species diversity and diversification rates with their phylogenetic history of host use. For these models, we had a single predictor variable.

##### Host‐use change

Host‐use change was a factor with three levels, indicating at each internal node if a host was gained, lost, or no change in use occurred. (Technically, gains and losses were identified as nodes at which the estimated host‐use state with the highest proportional likelihood different from that of the node's immediate ancestor.) This classification allowed us to test if host gains and losses tend to boost or throttle diversity.

**Figure 2:**
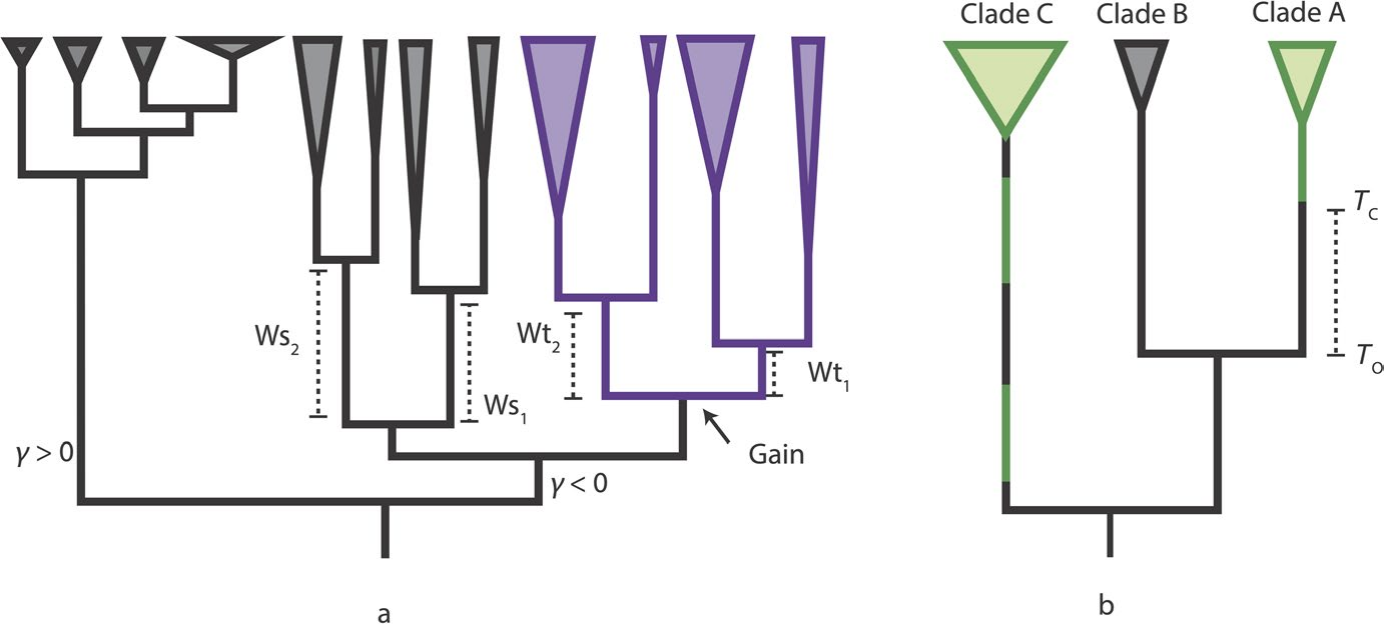
A schematic of diversification model covariates. (a) Response variables illustrated on hypothetical butterfly phylogeny where the width of triangles at tips is proportional to extant species richness. Branches in purple correspond to lineages with a novel host association. Waiting times for speciation following the evolution of a novel host association are indicated with Wt*_x_*. Speciation waiting times for the sister clade are indicated with Ws*_x_*. Slowing speciation rates correspond to a *γ* value <0. By contrast, accelerating speciation rates correspond to *γ* values >0. (b) Predictor variables illustrated on hypothetical plant phylogeny, green branches are used by a butterfly lineage, black branches are not; the width of triangles at tips is proportional to extant species richness; T_O_ is the stem age of a plant clade; T_C_ is the time at which that plant lineage was colonized by a butterfly lineage, and the difference between T_O_ and T_C_ is the early adoption statistic; clade C has a more volatile history of use by butterflies than clade A.

We used three response variables: (a) phylogenetically independent contrasts of speciation waiting times, (b) phylogenetically independent contrasts of extant species diversities, and (c) gamma statistic values (Pybus & Harvey, 2000). Here, we explain each (Figure 2).

##### Speciation waiting times

For each internal node in the butterfly phylogeny, we calculated the average length of the branches leading to its two immediate descendant nodes (i.e., the average waiting time for speciation). We then performed the same calculation for the focal node's sister node. The difference in average waiting times between sister nodes was used as a phylogenetically independent contrast of speciation rates. The sign and magnitude of these contrasts can show us how the gains and losses of novel host associations tend to immediately affect speciation rates. In short, this is just a comparison between two sister nodes, of the average time for subsequent lineage divergence.

##### Extant diversity

We calculated contrasts of extant nymphalid species richness in two ways, which we here refer to as exclusive and inclusive contrasts. For the exclusive contrasts, for each internal node in the butterfly phylogeny at which a host gain occurred, we calculated the total number of extant descendant species classified in genera that are known to continue to use the gained host. Then, in the focal node's sister clade, we counted all of the extant species in genera that do not use the novel host. For losses, we did the inverse; we counted extant descendant species that continued to not use the lost host and compared that count to the number of the descendant species of the sister node that continue to use the lost host. The differences between these extant diversities are a phylogenetically independent contrast of how the gains and losses of a host associations affect species diversity in the long term. For the inclusive contrasts, we compared *total* extant species diversities spanning each internal node; that is, we contrasted the extant diversity of a clade descended from an ancestor that gained or lost a new host, with the extant diversity of its sister clade, while ignoring whether or not extant taxa use (or do not use) that host (See Appendix [Link], [Link] for R scripts). This contrast is a more direct measure of the effect of a major host‐use change per se, as opposed to the effects of continued occupation of or exclusion from a particular adaptive space. To account for genera removed from the phylogeny during DEC reconstructions of host family use, these calculations were performed on the corresponding nodes on the complete tree (Wahlberg, 2006). For nodes at which no change in host use occurred, all descendants were contrasted in both the inclusive and exclusive diversity contrasts.

##### Gamma statistic

This is a measure of the degree to which speciation dynamics depart from expectations of an equal‐rates Markov model of phylogenetic branching (Pybus & Harvey, 2000). A value of zero corresponds to a constant diversification rate. Negative values indicate that speciation rates slow from the root to the tips, that is, there is an early burst of speciation (Pybus & Harvey, 2000), which is expected of an adaptive radiation. Positive values of the gamma statistic are difficult to interpret. They could indicate that diversification rates accelerate from the root the tips, that is, there is a late burst of speciation. Or they could simply be indicative of the so‐called “pull of the present”, an apparent late uptick in speciation rates due to the lag between speciation and extinction under a constant‐rate diversification regime. Hence, Pybus and Harvey (2000) recommend that positive values are ignored. We calculated the gamma statistic for all clades comprised of at least ten nymphalid genera.

#### 2.1.3 Linear model parameterization two—effects of ecological opportunity

We developed four indices for the scope of the ecological opportunity opened by the evolution of a novel host association and then used these as predictor variables to try an explain changes in butterfly diversity linked to evolutionary host gains. (a) *Host age* was the estimated phylogenetic age of the host‐plant taxon (family or order). Phylogenetic ages were taken from the TimeTree database (Kumar, Stecher, Suleski, & Hedges, 2017). All else being equal—in particular rates of speciation and ecological evolution—older plant lineages should be more diverse and have provided more time for herbivore diversification. (b) *The early adoption index* was the difference between the stem age of a host taxon and the time at which it began to be used as a host by a nymphalid lineage. Thus, it measures how quickly a particular butterfly lineage colonized a new host. If earlier colonizers are exposed to less competition and more open niche space, that could amount to greater ecological opportunity. (c) *Host diversity* was simply in the current species richness of the host family or order, according to the Catalogue of Life database (Roskov et al., 2018), accessed with the R package taxize (Chamberlain & Szöcs, 2013). (4) *Host volatility* was a count of how many times a particular host group was gained over the phylogeny, divided by the host age. Previous work has demonstrated that ancestral host associations condition the probability of host switching in extant populations (Futuyma, Keese, & Funk, 1995; Janz, Nyblom, & Nylin, 2001). Hence, many apparent host gains can be seen as the re‐expression of a latent phenotype, and the phylogenetic history of the use of some host taxa can appear quite volatile. By contrast some, hosts are seldom gained and lost. Colonization of such hosts may represent more novel niche transformations and greater ecological opportunities.

#### 2.1.4 Model fitting

To repeat, we sought to address two questions: Do host‐use gains tend to increase diversity and if so, do novel associations corresponding to greater ecological opportunities spark greater diversity gains? For the first question, we looked at how host‐use gains and losses affected extant species diversity, speciation waiting times, and the overall dynamics of diversification (as measured with the gamma statistic). For the second question, we looked at the same response variables, but only for host gains, and tried to explain the variation in the response variables with several indices of the breadth of ecological opportunity afforded by a novel host group. Two of the response variables—speciation waiting times and extant species diversities—were phylogenetically independent contrasts. Therefore, we could use standard linear modeling methods to estimate the fixed effects of the predictor variables on their variance. (We used the built‐in lm R function.) To account for uncertainty in the DEC* reconstructions, we weighted each model covariate with a vector of the proportional probabilities for each estimated ancestral host‐use state. For these models, as is standard for models of phylogenetically independent contrasts, we forced the regression to pass through the origin.

Values of the third response variable, the gamma statistic, were not phylogenetically independent. To account for this, we fit linear mixed models in which phylogenetic relatedness between nodes was expressed using a pedigree structure and included this as a random effect with the Bayesian approach implemented in MCMCglmm (Hadfield, 2010). Analyses consisted of 1,000,000 MCMC iterations with a thinning interval of 100. We used the Geweke diagnostic to confirm that we had sampled sufficiently from the stationary distribution (see Tables S1–S16 for all model results). To correct for bias in gamma statistic estimates due to incomplete sampling of nymphalid branches, we weighted each empirical gamma statistic by its distance in standard deviations from the mean gamma statistic value estimated from 100 simulations under a null birth–death model, as implemented by the MCCR test (Pybus & Harvey, 2000) in the R package phytools (Revell, 2012). Note that we incorporated these weights by using the “mev” argument of MCMCglmm, which is intended to take a vector of effect size variances for a meta‐analysis (See Appendix S12 for full model specifications).

#### 2.1.5 Explicit state‐dependent early burst models

As previously mentioned, simulation studies have shown that DEC models appear to accurately estimate the ancestral states of multi‐state discrete traits—as long as trait states do not strongly affect species diversification rates. Violation of that assumption can bias reconstructions (Maddison, Midford, & Otto, 2007). Several models have been developed that can estimate the ancestral states of a discrete trait, while explicitly accounting for state‐dependent variation in speciation and extinction rates (e.g., see Goldberg, Lancaster, & Ree, 2011). Unfortunately, it is currently not feasible to fit such models to traits with as many states as host use in nymphalids. To work around that constraint, and as a complement to our DEC‐based analyses, we fit explicit state‐dependent early burst models to the phylogenetic history of binary (use or nonuse) traits for each nymphalid host taxon, using the fitDiscrete function in the R package geiger (Harmon, Weir, Brock, Glor, & Wendell, 2008). Then, for each host taxon, we compared the fit of the early burst model to a model with constant branching and extinction. Comparisons were made with likelihood ratio tests, using the R package extRemes (Gilleland & Katz, 2016). To be clear, both our main DEC‐based analyses and the state‐dependent early burst analyses are subject to known biases, but these biases are different.

### 2.2 Species‐level analyses

To complement the main genus‐level analysis, we repeated a subset of tests at the level of species. For these tests, we looked only at the use of host‐plant families and excluded from consideration any family used by fewer than five nymphalid species. We also excluded any nymphalid species without host‐use data, or that used only one of the excluded host‐plant families. To keep tractable DEC estimates of ancestral host use, for nymphalid subtrees with 10 or more extant host‐plant families, we capped the maximum diet breadth at five families. This left us with associations between 57 host‐plant families and 1,189 nymphalid species, apportioned over 16 analyzable subtrees. We looked only at inclusive contrasts of extant species diversity, and models explaining variation in gamma statistics were not weighted by MCCR tests.

## 3 RESULTS

### 3.1 Genus‐level analyses

**Figure 3:**
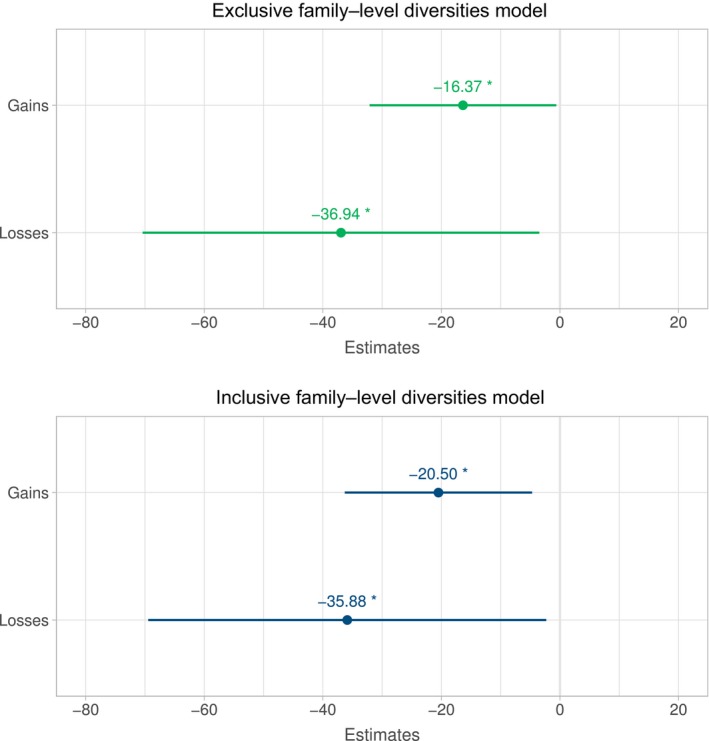
Plots of estimated coefficients for significant effects of host gains and losses in the family‐level model shown along with 95% confidence interval

Looking at host‐plant families, both host‐use gains and losses were negatively correlated with extant diversity. As explained above, we calculated contrasts of diversity in two ways for gains. In the first, which we refer to as exclusive contrasts, we compared the number of species in a focal clade that use a particular host group to the number of species in the sister clade that do not use that host group. For losses, we did the inverse. In the second, which we refer to as inclusive contrasts, we compared the total extant species diversity (regardless of current host use) descended from an ancestor that gained a host group to the total species diversity of its sister group. In the exclusive contrasts family‐level model, nodes with a host‐use gain had on average 16.37 fewer extant descendant species than their sister nodes (*p*‐value .042), while nodes with a host‐use loss had on average 36.94 fewer extant descendant species than their sister nodes (*p*‐value: .031). In the inclusive‐contrast family‐level model, nodes with a host‐use gain had on average 20.50 fewer extant descendant species than their sister node (*p*‐value .011), while nodes with a host‐use loss had on average 35.88 fewer extant species than their sister node (*p*‐value: .036; see Table 1 and Figure 3). To put those figures in perspective, the average summed species diversity of the focal and sister clades involved in a contrast was 69.37. Note that in both cases, the estimated magnitude of the effects on diversity was greater for losses. Because of the paucity of internal nodes at which family‐level host‐use changes were reconstructed on nymphalid subtrees, we were unable to estimate the effects of ecological opportunity proxies on variation in gamma statistic values or speciation waiting times. We found no significant effects from any of the ecological opportunity proxies on variation in extant diversities (Tables S8–S16).

**Table 1:** Values for host‐use change as a function of species diversity (family models)

	Coefficient	*p*‐Value
Exclusive gain	−16.4	.042
Exclusive loss	−36.9	.031
Inclusive gain	−20.5	.011
Inclusive loss	−35.9	.036

**Figure 4:**
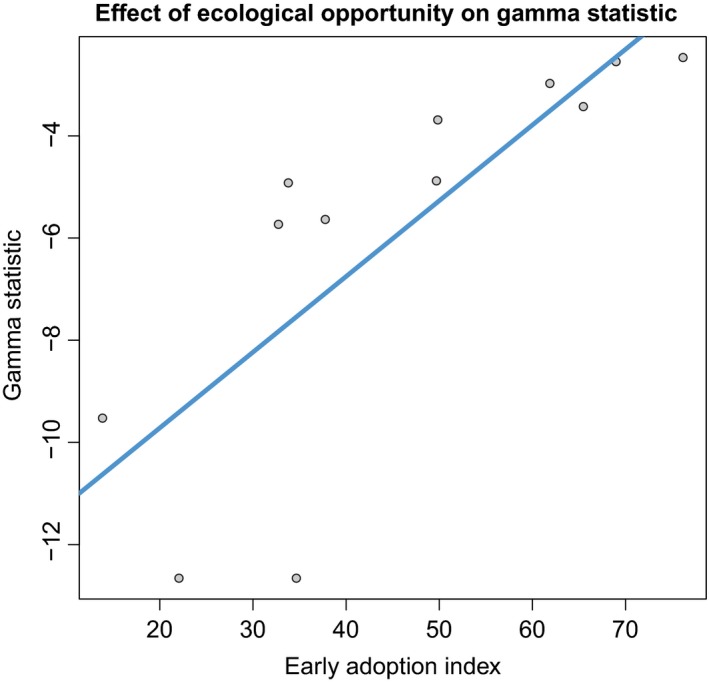
Simplified order‐level model of effect of early adoption index on gamma statistic values. This model did not account for phylogenetic non‐independence. The plot shows that and early‐burst of diversification is more pronounced when a butterfly lineage colonized a novel host‐plant lineage earlier in the latter's independent evolutionary history

Looking at host‐plant orders, host‐use change did not have any significant effects on diversity. Likewise, the proxies for the magnitude of ecological opportunity were mostly uncorrelated with diversity dynamics (Tables S1–S8). There was one exception; gamma statistic values were positively correlated with early adoption index values (estimated coefficient: 0.22, *p*‐value: .012; See Figure 4; Appendix S13 shows the distribution of gamma statistic values.)

A state‐dependent early burst model of phylogenetic branching was a better fit than a constant diversification rate model for just seven of 128 host‐plant families (each with *p*‐value < .001): Apiaceae, Apocynaceae, Boraginaceae, Cyperaceae, Euphorbiaceae, Rosaceae and Verbenaceae. For full results, see Table S23.

### 3.2 Species‐level analyses

Results of the nymphalid species‐level analyses add nuance. As in the genus‐level analyses, both host gains and losses tended to be linked to decreasing in extant species richness (gains coefficient: −9.782, *p*‐value: .017; losses coefficient: −5.379, *p*‐value: .44). Major host shifts tend to have negative long‐term effects on diversity. And as in the genus‐level analyses, we found a negative correlation between extant diversity contrasts and the early adoption index (coefficient: −0.57, *p*‐value: <.001); if a nymphalid lineage colonized a novel host group early in its independent evolutionary history, that tended to reduce the negative effects on extant species richness. Analysis of the nymphalid species‐level data also revealed a positive correlation between extant nymphalid diversity and host‐plant family age (coefficient: 0.56, *p*‐value: <.001). For full results of the species‐level models, see Tables S17–S22.

## 4 DISCUSSION

Does the Escape and Radiate Hypothesis predict phylogenetic patterns of host‐use and species diversity? For the most part, this does not seem to be the case. We find no support for the broadest prediction that the evolution of major new host associations tends to boost herbivorous insect diversity over long phylogenetic time scales. To the contrary, we find evidence that novel associations tend to decrease extant butterfly species richness. It could be that major evolutionary changes in host use are caused by major declines in fitness on ancestral hosts. Such declines could be due to a variety of factors including increased competition for diminished host‐plant resources, the evolution of novel host defenses, the invasion of host herbivore assemblages by new species, and changes in natural enemy communities (Bird, Kaczvinsky, Wilson, & Hardy, 2019; Kenis et al., 2009; Segraves & Anneberg, 2016). Regardless of the cause, if major evolutionary changes in herbivore diet are sparked by such calamities, we might expect such changes to be linked to long‐term decreases in herbivorous insect species diversity. With reduced performance on ancestral hosts, and likely marginal performance on new hosts, the growth rates and effective sizes of herbivorous insect populations could shrink along with their geographic and climatic niche ranges. This could increase the odds of extinction and decrease the odds of speciation. Thus, major evolutionary shifts in the diets of butterflies could mark ecological crashes more than ecological opportunities.

Alternatively, it is possible that major new host associations are, in fact, the realization of ecological opportunities, but such opportunities do not tend to promote speciation of nymphalids in the long term. We found evidence of early burst diversification linked to the evolution of the use of several specific plant families. This suggests that coevolutionary adaptive radiation may indeed have played a role in the diversification of herbivorous insects. But such events might have been relatively rare in the history of nymphalids, and short‐term increases in speciation might come with tight constraints on long‐term diversity.

The Escape and Radiate Hypothesis can also be used to predict that a novel host association should affect species diversity in a way that is proportional to the scope of ecological opportunity the new host group offers. Two of our indices of ecological opportunity could explain some of the variation in our butterfly diversification variables. First, we found that gamma statistic values were more negative—indicating an earlier burst of diversification (Gavrilets & Losos, 2009)— when butterfly lineages colonized a novel host taxon earlier in the latter's independent evolutionary history. This is consistent with our assumption of emptier niche space earlier in a plant lineage's evolutionary history. (This in turn is based on the assumption that the origins of major plant clades tend to be linked to events which release plants from their previous consumers.) Second, we found that the effect of evolving to use a novel host‐plant family on the extant species diversity of nymphalid clades depended on the age of the novel host group, with older hosts having more positive effects on nymphalid diversity. This is consistent with our assumption that, all else being equal, older host taxa should tend to be more diverse and correspond to greater ecological opportunities. Each of these effects could be interpreted as evidence in support of the notion that the scope of ecological opportunity is a key limitation on herbivorous insect diversity. Nevertheless, since the estimated overall effect of host‐use gains on butterfly species diversity tends to be negative, it would seem that broader ecological opportunities on novel hosts have tended to soften the blow of a major host shift, rather than fan the flames of explosive speciation.

Of course, this study has several limitations. Most importantly, we worked within the limits of the current models for reconstructing the phylogenetic history of a complex trait like host use. As mentioned above, it is currently not possible to reconstruct the ancestral states of a highly multi‐state discrete trait while accounting for state‐dependent variation in speciation and extinction rates (Maddison et al., 2007). To work around this constraint, we took two approaches, each with its own shortcoming. In the first, we reconstructed host‐use evolution with state‐independent diversification DEC* models and then performed *post hoc* analyses of diversity patterns. In the second, we fit explicitly state‐dependent models of early burst diversification for nymphalid genera, but on a series of binary host‐use characters. The shortcoming of the first approach is the assumption of state‐independent diversification. The shortcoming of the second is that when a multi‐state trait such as host use is modeled as series of binary traits we tend to reconstruct ancestors without hosts (Hardy, 2017). The picture of nymphalid diversification that emerges—in which coevolutionary adaptive radiation plays a relatively small role, and major host‐use shifts may more often denote times of ecological calamity than opportunity—appears at least to be consistent across approaches. But until more powerful comparative methods have been developed, it will be difficult to make stronger conclusions. We were also limited by crude and indirect proxies of the ecological opportunity attached to a novel host association. As we learn more about ecological speciation and community assembly in herbivorous insects, we may find ways of more accurately quantifying historical ecological opportunity.

At first blush, the results presented here may appear to contradict those of Hardy and Otto (2014), who found that butterfly lineages that transition more frequently between monophagy and polyphagy tend to speciate more rapidly. This is especially problematic if the rate of diet‐breadth oscillation is used as an index of the overall rate of host‐use evolution, or of the odds that a butterfly lineage will gain or lose a novel host association. The apparent incongruence between this study and that one could be a sign that our inferences of ancestral host use and diversification dynamics have been warped by the shortcomings in our models. Alternatively, it could mean that the rate of transition between monophagy and polyphagy is not a good index of the overall rate of host‐use evolution. Major host groups could be mostly gained and lost by generalist lineages; in that case, rapid transitions between monophagy and polyphagy could be linked to rapid speciation, even if major host‐use changes tend to depress diversity. Other explanations could be fashioned. In sum, the surprising evolutionary patterns we find here could indicate that our approach was inadequate, or it could simply reflect that the evolutionary history of host‐use and speciation in butterflies is complex and poorly understood.

We framed our analyses as tests of the Escape and Radiate Hypothesis. In our view, this is consistent with its current usage and connotations, but to be sure, those connotations have evolved since Ehrlich and Raven (1964). In fact, its earliest formulations were vague enough so as to make it neigh impossible to falsify. Subsequent work (e.g., Fordyce, 2010; Janz & Nylin, 2008; Winkler, Mitter, & Scheffer, 2009) sharpened its predictions while also blurring its attributions. Hence, although our findings do not support that major host colonization events spark adaptive radiation of herbivorous insects, one could argue that this is within the realm of expectations under the Escape and Radiate Hypothesis. (In that case, the Escape and Radiate Hypothesis might not be very useful for understanding the macro‐evolution of herbivorous insects.)

In sum, our tests yield evidence against a key prediction of the Escape and Radiate Hypothesis: that the evolution of major new host associations will tend to boost the species diversity of herbivorous insect lineages. We find some evidence that the scope of ecological opportunity afforded by a novel host association governs its effects on diversification dynamics. But since the overall effect of a major new association appears to be negative, it seems that large new opportunities function to primarily diminish negative effects, rather than to inflate positive effects. To be sure, this evidence is conditioned on the adequacy of the models we have used to infer ancestral host use and diversification dynamics, but it is consistent with another recent comparative phylogenetic study finding that patterns in the networks of evolutionary associations between butterflies and their host plants are inconsistent with coevolutionary adaptive radiation (Braga, Guimarães, Wheat, Nylin, & Janz, 2018). For half a century, the Escape and Radiate Hypothesis has inspired evolutionary ecologists' explanations of the incredible diversity of herbivorous insects. And as researchers continue to build our capacity to model herbivorous insect diversification, we will be able to perform more powerful tests of the Escape and Radiate Hypothesis's predictions. In the meantime, from the evidence against it, we may find inspiration for alternative explanations.

## CONFLICT OF INTEREST

None declared.

## AUTHOR CONTRIBUTION

CK performed most of the scripting; CK and NBH jointly conceived of the project and helped draft the manuscript. All authors gave final approval for publication and agree to be held accountable for the work performed therein.

## Data Availability

All data used in this study are from published studies or open access databases. All data for this project have been included in supplements or is drawn from published works.

## References

[ece36116-bib-0001] Agrawal, A. A. (2007). Macroevolution of plant defense strategies. Trends in Ecology and Evolution, 22(2), 103–109. 10.1016/j.tree.2006.10.012 17097760

[ece36116-bib-0002] Althoff, D. M. , Segraves, K. A. , & Johnson, M. T. J. (2014). Testing for coevolutionary diversification: Linking patter with process. Trends in Ecology and Evolution, 29(2), 82–89. 24314843 10.1016/j.tree.2013.11.003

[ece36116-bib-0003] Bird, G. , Kaczvinsky, C. , Wilson, A. , & Hardy, N. B. (2019). When do herbivorous insects compete? A phylogenetic meta‐analysis. Ecology Letters, 22(5), 875–883. 10.1111/ele.13245 30848045

[ece36116-bib-0004] Braby, M. F. , & Trueman, J. W. H. (2006). Evolution of larval host plant associations and adaptive radiation in pierid butterflies. Journal of Evolutionary Biology, 19(5), 1677–1690. 10.1111/j.1420-9101.2006.01109.x 16910997

[ece36116-bib-0005] Braga, M. P. , Guimarães, P. R. , Wheat, C. W. , Nylin, S. , & Janz, N. (2018). Unifying host‐associated diversification processes using butterfly‐plant networks. Nature Communications, 9, 5155 10.1038/s41467-018-07677-x PMC6279759 30514925

[ece36116-bib-0006] Chamberlain, S. A. , & Szöcs, E. (2013). taxize: Taxonomic search and retrieval in R. F1000Research, 2, 191 10.12688/f1000research.2-191.v1 24555091 PMC3901538

[ece36116-bib-0007] Ehrlich, P. R. , & Raven, P. H. (1964). Butterflies and Plants: A study in coevolution. Evolution, 18(4), 586–608. 10.2307/2406212

[ece36116-bib-0008] Farrell, B. D. , Dussourd, D. E. , & Mitter, C. (1991). Escalation of plant defense: Do latex and resin canals spur plant diversification? American Naturalist, 138(4), 881–900. 10.1086/285258

[ece36116-bib-0009] Farrell, B. D. , & Mitter, C. (1994). Adaptive radiation in insects and plants: Time and opportunity. American Zoologist, 34(1), 57–69. 10.1093/icb/34.1.57

[ece36116-bib-0010] Fordyce, J. A. (2010). Host shifts and evolutionary radiations of butterflies. Proceedings of the Royal Society B: Biological Sciences, 277, 3735–3743. 10.1098/rspb.2010.0211 PMC2992698 20610430

[ece36116-bib-0011] Futuyma, D. J. , & Agrawal, A. A. (2009). Macroevolution and the biological diversity of plants and herbivores. Proceedings of the National Academy of Sciences of the United States of America, 106(43), 18054–18061. 10.1073/pnas.0904106106 19815508 PMC2775342

[ece36116-bib-0012] Futuyma, D. J. , Keese, M. C. , & Funk, D. J. (1995). Genetic constraints on macroevolution: The evolution of host affiliation in the leaf beetle genus *Ophraella* . Evolution, 49(5), 797–809. 10.2307/2410403 28564882

[ece36116-bib-0013] Futuyma, D. J. , & Mitter, C. (1996). Insect‐plant interactions: The evolution of component communities. Philosophical Transactions of the Royal Society B: Biological Sciences, 351, 1361–1366.

[ece36116-bib-0014] García Morales, M. , Denno, B.D. , Miller, D.R. , Miller, G.L. , Ben-Dov, Y. , & Hardy, N.B. , (2016). ScaleNet: a literature-based model of scale insect biology and systematics. Database, 2016. 10.1093/database/bav118 PMC4747323 26861659

[ece36116-bib-0015] Gavrilets, S. , & Losos, J. B. (2009). Adaptive radiation: Contrasting theory with data. Science, 323(5915), 732–738. 10.1126/science.1157966 19197052

[ece36116-bib-0016] Gilleland, E. , & Katz, R. W. (2016). extRemes 2.0: An extreme value analysis package in R. Journal of Statistical Software, 72(8), 1–31.

[ece36116-bib-0017] Goldberg, E. , Lancaster, L. T. , & Ree, R. H. (2011). Phylogenetic inference of reciprocal effects between geographic range evolution and diversification. Systematic Biology, 60(4), 451–465. 10.1093/sysbio/syr046 21551125

[ece36116-bib-0018] Hadfield, J. (2010). MCMC methods for Multi‐Response Generalized Linear Mixed Models: The MCMCglmm R package. Journal of Statistical Software, 33(2), 1–22. 10.1111/j.1365-2621.1992.tb08045.x 20808728

[ece36116-bib-0019] Hamm, C. A. , & Fordyce, J. A. (2015). Patterns of host plant utilization and diversification in the brush‐footed butterflies. Evolution, 69(3), 589–601. 10.1111/evo.12593 25546268

[ece36116-bib-0020] Hardy, N. B. (2017). Do plant‐eating insect lineages pass through phases of host‐use generalism during speciation and host switching? Phylogenetic evidence. Evolution, 71(8), 2100–2109. 10.1111/evo.13292 28654210

[ece36116-bib-0021] Hardy, N. B. , Gullan, P. J. , & Hodgson, C. J. (2008). A subfamily-level classification of mealybugs (Hemiptera: Pseudococcidae) based on integrated molecular and morphological data. Systematic Entomology, 33(1), 51–71.

[ece36116-bib-0022] Hardy, N. B. , & Otto, S. P. (2014). Specialization and generalization in the diversification of phytophagous insects: Tests of the musical chairs and oscillation hypotheses. Proceedings of the Royal Society B: Biological Sciences, 281, 20132960 10.1098/rspb.2013.2960 PMC4213601 25274368

[ece36116-bib-0023] Harmon, L. J. , Weir, J. T. , Brock, C. D. , Glor, R. E. , & Wendell, C. (2008). GEIGER: Investigating evolutionary radiations. Bioinformatics, 24(1), 129–131. 10.1093/bioinformatics/btm538 18006550

[ece36116-bib-0024] Hembry, D. H. , Yoder, J. B. , & Goodman, K. R. (2014). Coevolution and the diversification of life. American Naturalist, 184(4), 425–438. 10.1086/677928 25226178

[ece36116-bib-0025] Janz, N. (2011). Ehrlich and Raven revisited: Mechanisms underlying codiversification of plants and enemies. Annual Review of Ecology, Evolution, and Systematics, 42, 71–89. 10.1146/annurev-ecolsys-102710-145024

[ece36116-bib-0026] Janz, N. , Nyblom, K. , & Nylin, S. (2001). Evolutionary dynamic of host‐plant specialization: A case study of the tribe Nymphalini. Evolution, 55, 783–796. 10.1554/0014-3820(2001)055[0783:EDOHPS]2.0.CO;2 11392396

[ece36116-bib-0027] Janz, N. , & Nylin, S. (2008). The oscillation hypothesis of host‐plant range and speciation In Tillman K. (Eds.), Specialization, speciation, and radiation: The evolutionary biology of herbivorous insects (pp. 203–215). Berkeley, CA: University of California Press.

[ece36116-bib-0028] Kenis, M. , Auger‐Rozenberg, M. A. , Roques, A. , Timms, L. , Péré, C. , Cock, M. J. W. , … Lopez‐Vaamonde, C. (2009). Ecological effects of invasive alien insects. Biological Invasions, 11(1), 21–45. 10.1007/s10530-008-9318-y

[ece36116-bib-0029] Kergoat, G. J. , Silvain, J.‐F. , Delobel, A. , Tuda, M. , & Anton, K.‐W. (2007). Defining the limits of taxonomic conservatism in host – plant use for phytophagous insects: Molecular systematics and evolution of host – plant associations in the seed‐beetle genus *Bruchus* Linnaeus (Coleoptera: Chrysomelidae: Bruchinae). Molecular Phylogenetics and Evolution, 43(1), 251–269. 10.1016/j.ympev.2006.11.026 17276089

[ece36116-bib-0030] Kumar, S. , Stecher, G. , Suleski, M. , & Hedges, S. B. (2017). TimeTree: A resource for timelines, timetrees, and divergence times. Molecular Biology and Evolution, 34(7), 1812–1819. 10.1093/molbev/msx116 28387841

[ece36116-bib-0031] Liscombe, D. K. , Macleod, B. P. , Loukanina, N. , Nandi, O. I. , & Facchini, P. J. (2005). Evidence for the monophyletic evolution of benzylisoquinoline alkaloid biosynthesis in angiosperms. Molecular Biology and Evolution, 66(11), 1374–1393. 10.1016/j.phytochem.2005.04.029 15925393

[ece36116-bib-0032] Losos, J. B. , & Mahler, D. L. (2010). Adaptive radiation: The interaction of ecological opportunity, adaptation, and speciation In Bell M. A., Futuyma D. J., Eanes W. F., Levinton J. S., Bell M. A., Futuyma D. J., & Eanes W. F. (Eds.), Evolution since Darwin: The first 150 years (pp. 381–420). Sunderland, MA: Sinauer Associates.

[ece36116-bib-0033] Maddison, W. P. , Midford, P. E. , & Otto, S. P. (2007). Estimating a binary character's effect on speciation and extinction. Systematic Biology, 56(5), 701–710. 10.1080/10635150701607033 17849325

[ece36116-bib-0034] Massana, K. A. , Beaulieu, J. M. , Matzke, N. J. , & O'Meara, B. C. (2015). Non‐null effects of the null range in biogeographic models: Exploring parameter estimation in the DEC model. bioRxiv, 026914 10.1101/026914

[ece36116-bib-0035] Matzke, N. J. (2013). BioGeoBEARS: BioGeography with Bayesian (and likelihood) evolutionary analysis in R scripts. R package, version 0.2.1. http://CRAN.R-project.org/package=BioGeoBEARS

[ece36116-bib-0036] Matzke, N. J. (2014). Model selection in historical biogeography reveals that founder‐event speciation is a crucial process in Island Clades. Systematic Biology, 63(6), 951–970. 10.1093/sysbio/syu056 25123369

[ece36116-bib-0037] Peterson, D. A. , Hardy, N. B. , & Normark, B. B. (2016). Micro‐and macroevolutionary trade‐offs in plant‐feeding insects. American Naturalist, 188(6), 640–650. 10.1086/688764 27860513

[ece36116-bib-0038] Pybus, O. G. , & Harvey, P. H. (2000). Testing macro‐evolutionary models using incomplete molecular phylogenies. Proceedings of the Royal Society B: Biological Sciences, 267, 2267–2272. 10.1098/rspb.2000.1278 PMC1690817 11413642

[ece36116-bib-0039] R Core Team (2019). R: A language and environment for statistical computing. Vienna, Austria: R Foundation for Statistical Computing.

[ece36116-bib-0040] Revell, L. J. (2012). phytools: An R package for phylogenetic comparative biology (and other things). Methods in Ecology and Evolution, 3(2), 217–223. 10.1111/j.2041-210X.2011.00169.x

[ece36116-bib-0041] Robinson, G. S. , Ackery, P. R. , Kitching, I. J. , Beccaloni, G. W. , & Hernández, L. M. (2010). HOSTS‐a database of the World's Lepidopteran hostplants. London, UK: Natural History Museum.

[ece36116-bib-0042] Roskov, Y. , Abucay, L. , Orrell, T. , Nicolson, D. , Bailly, N. , Kirk, P. M. , …, van Zarucchi, J. P. L. (2018). Species 2000 & ITIS Catalogue of life, 2018 Annual checklist. Retrieved from https://www.catalogueoflife.org/annual-checklist/2018

[ece36116-bib-0043] Schluter, D. (2000). The ecology of adaptive radiation. Oxford, UK: Oxford University Press.

[ece36116-bib-0044] Segraves, K. A. , & Anneberg, T. J. (2016). Species interactions and plant polyploidy. American Journal of Botany, 103(7), 1326–1335. 10.3732/ajb.1500529 27370313

[ece36116-bib-0045] Suchan, T. , & Alvarez, N. (2015). Fifty years after Ehrlich and Raven, is there support for plant–insect coevolution as a major driver of species diversification? Entomologia Experimentalis et Applicata, 157(1), 98–112.

[ece36116-bib-0046] Thompson, J. N. (1989). Concepts of coevolution. Trends in Ecology and Evolution, 4(6), 179–183. 10.1016/0169-5347(89)90125-0 21227347

[ece36116-bib-0047] Wahlberg, N. (2006). That awkward age for butterflies: Insights from the age of the butterfly subfamily Nymphalinae (Lepidoptera: Nymphalidae). Systematic Biolgoy, 55(5), 703–714. 10.1080/10635150600913235 16952908

[ece36116-bib-0048] Wheat, C. W. , Vogel, H. , Wittstock, U. , Braby, M. F. , Underwood, D. , & Mitchell‐Olds, T. (2007). The genetic basis of a plant insect coevolutionary key innovation. Proceedings of the National Academy of Sciences of the United States of America, 104(51), 20427–20431. 10.1073/pnas.0706229104 18077380 PMC2154447

[ece36116-bib-0049] Wink, M. (2003). Evolution of secondary metabolites from an ecological and molecular phylogenetic perspective. Phytochemistry, 64(1), 3–19. 10.1016/S0031-9422(03)00300-5 12946402

[ece36116-bib-0050] Wink, M. , & Mohamed, G. I. A. (2003). Evolution of chemical defense traits in the Leguminosae: Mapping of distribution patterns of secondary metabolites on a molecular phylogeny inferred from nucleotide sequences of the rbcL gene. Biochemical Systematics and Ecology, 31(8), 897–917. 10.1016/S0305-1978(03)00085-1

[ece36116-bib-0051] Winkler, I. S. , Mitter, C. , & Scheffer, S. J. (2009). Repeated climate‐linked host shifts have promoted diversification in a temperate clade of leaf‐mining flies. Proceedings of the National Academy of Sciences of the United States of America, 106(43), 18103–18108. 10.1073/pnas.0904852106 19805134 PMC2775340

[ece36116-bib-0052] Yoder, J. B. , Clancey, E. , Des Roches, S. , Eastman, J. M. , Gentry, L. , Godsoe, W. , … Harmon, L. J. (2010). Ecological opportunity and the origin of adaptive radiations. Journal of Evolutionary Biology, 23(8), 1581–1596. 10.1111/j.1420-9101.2010.02029.x 20561138

