# Supplementary figures and images for: Do major host shifts spark diversification in butterflies?
Source: Ecol Evol. 2020 Feb 26;10(8):3636–46. doi: 10.1002/ece3.6116 (PMC7160180; doi:10.1002/ece3.6116)

Order-Level DEC\* Reconstruction

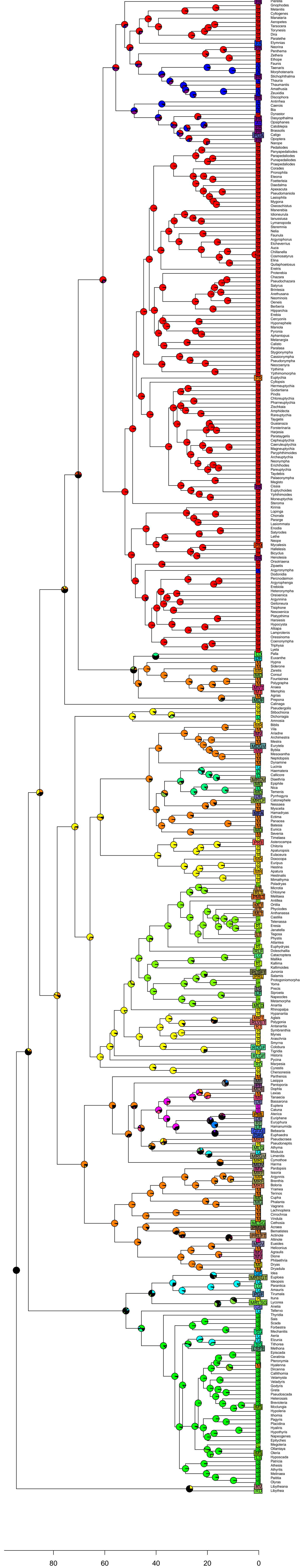

Supplement: Supplementary file 2 [file ECE3-10-3636-s003.pdf]

Family-Level Subtree 2 DEC\* Reconstruction

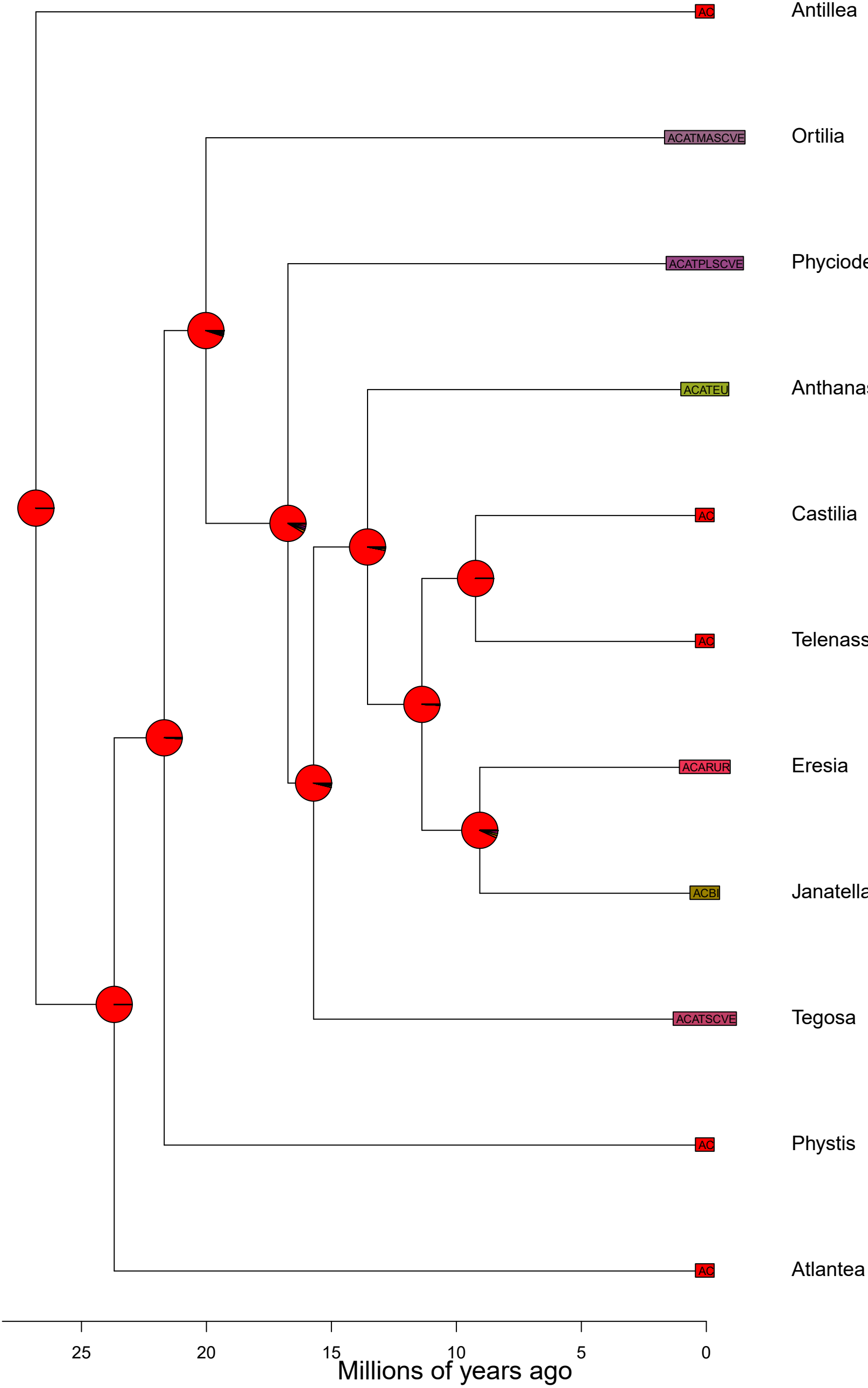

Supplement: Supplementary file 3 [file ECE3-10-3636-s004.pdf]

Family-Level Subtree 3 DEC\* Reconstruction

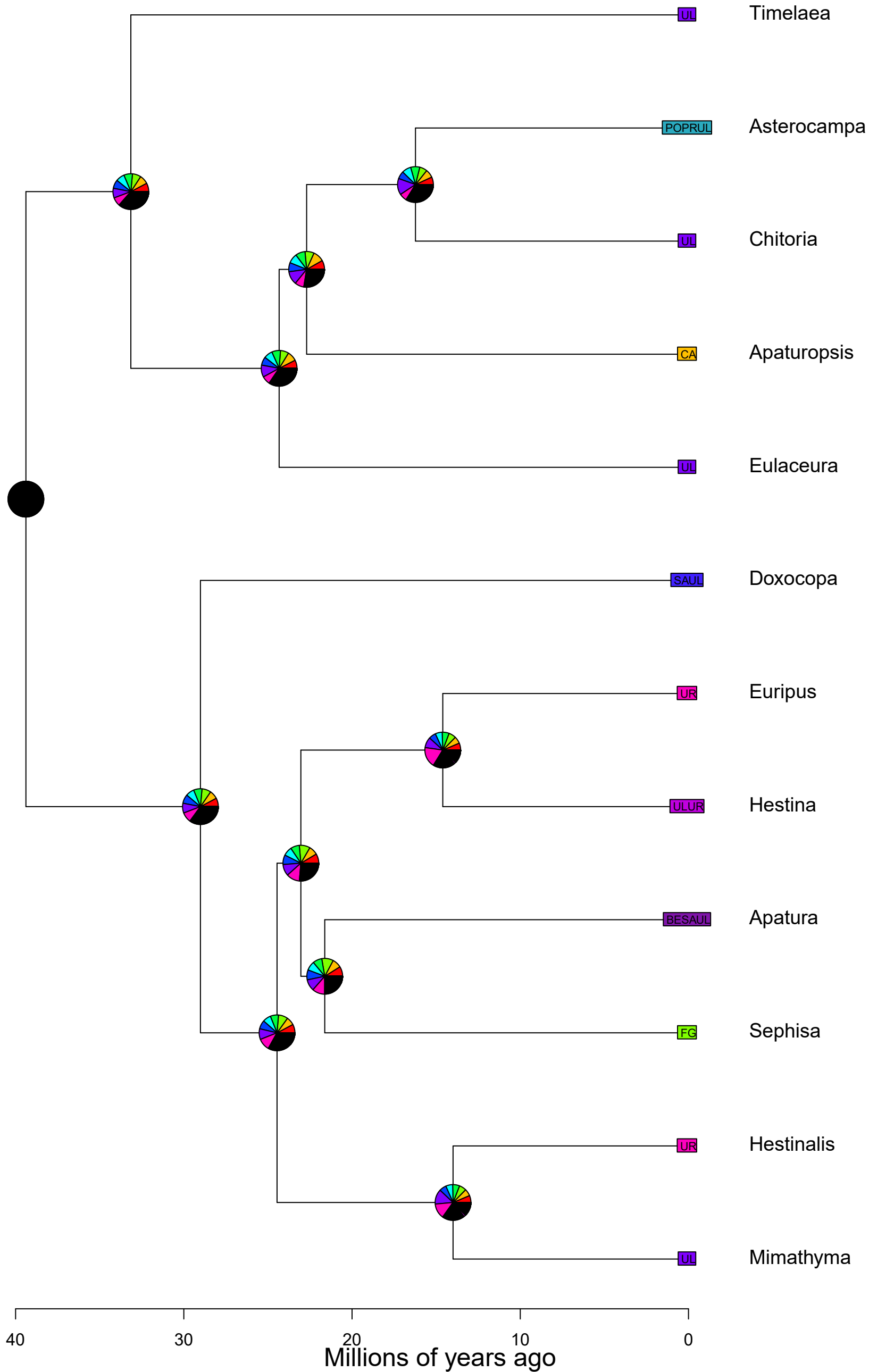

Supplement: Supplementary file 4 [file ECE3-10-3636-s005.pdf]

Family-Level Subtree 4 DEC\* Reconstruction

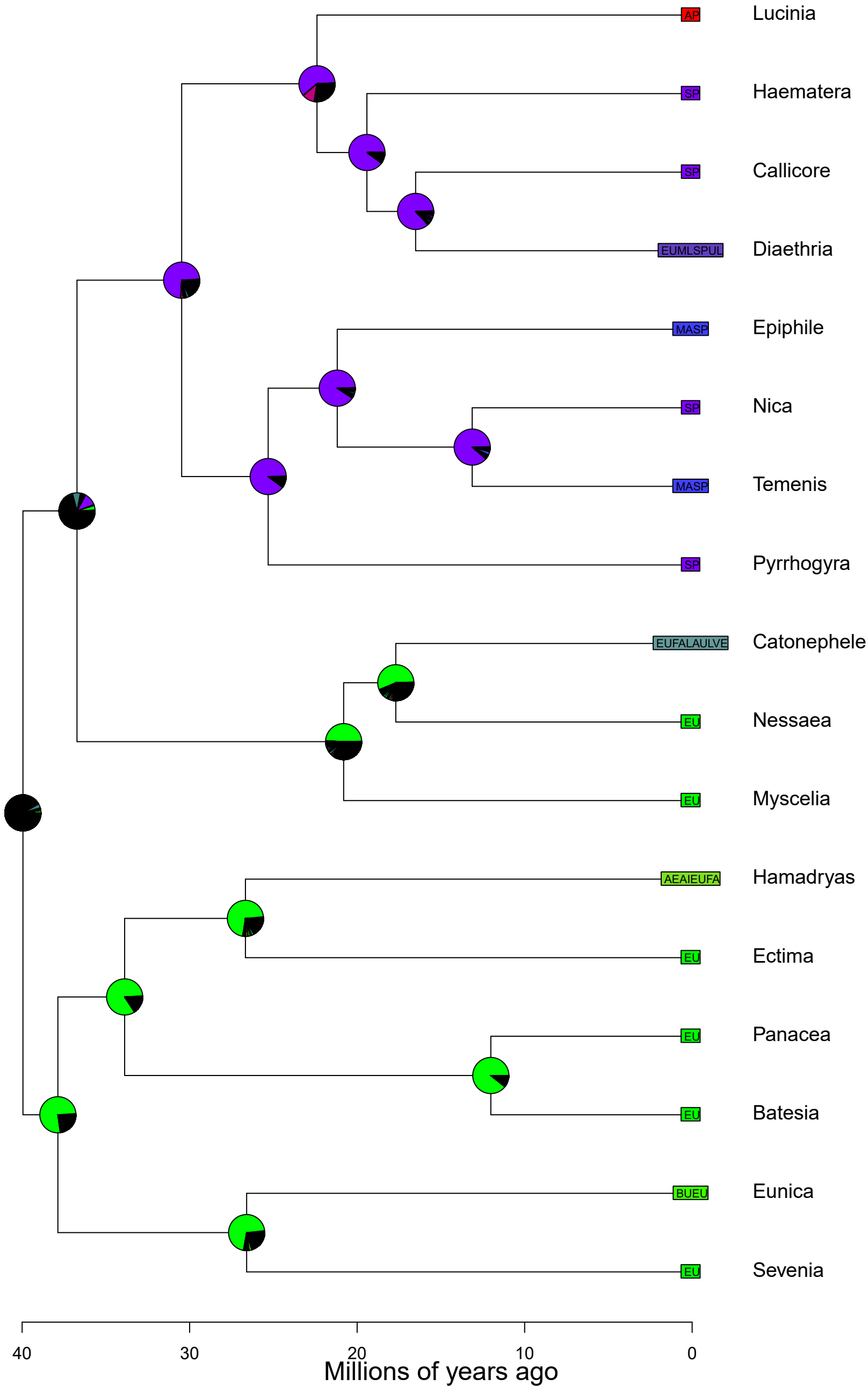

Supplement: Supplementary file 5 [file ECE3-10-3636-s006.pdf]

Family-Level Subtree 5 DEC\* Reconstruction

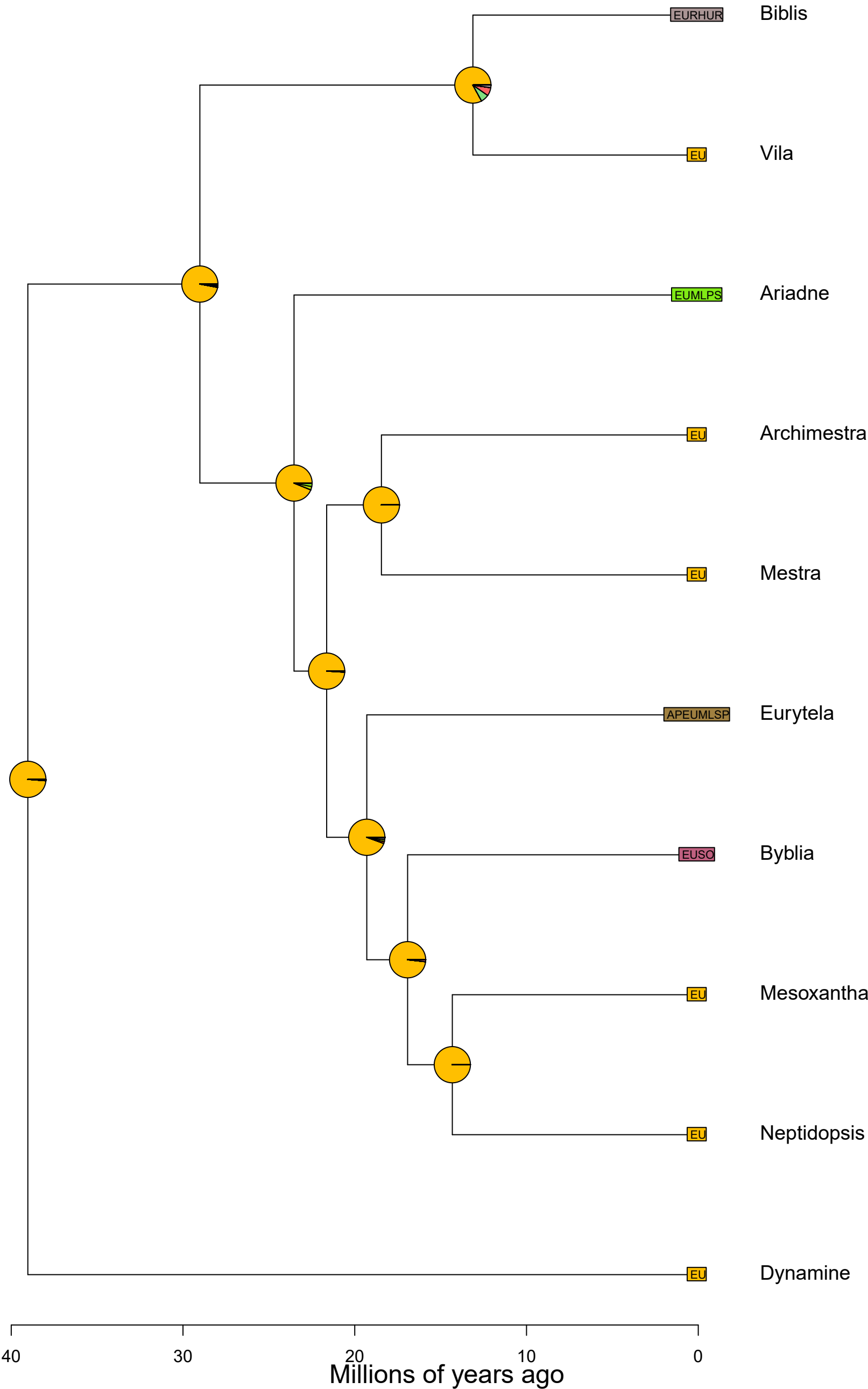

Supplement: Supplementary file 6 [file ECE3-10-3636-s007.pdf]

Family-Level Subtree 6 DEC\* Reconstruction

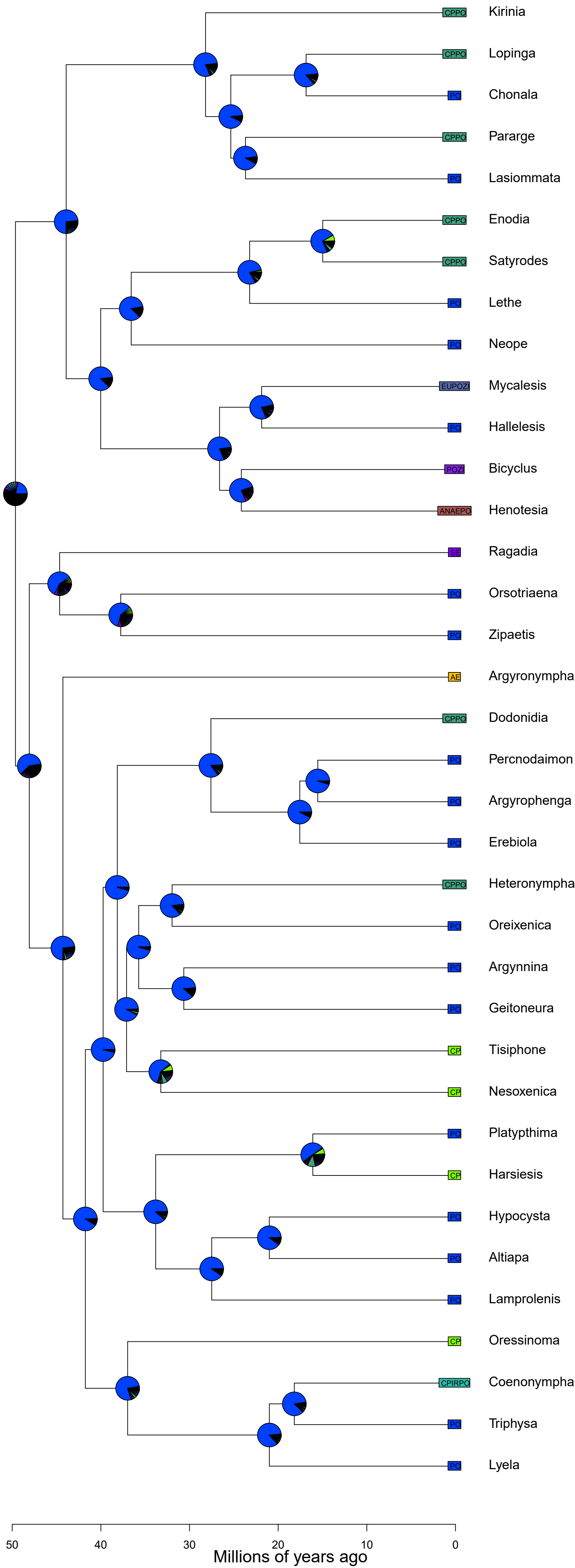

Supplement: Supplementary file 7 [file ECE3-10-3636-s008.pdf]

Family-Level Subtree 7 DEC\* Reconstruction

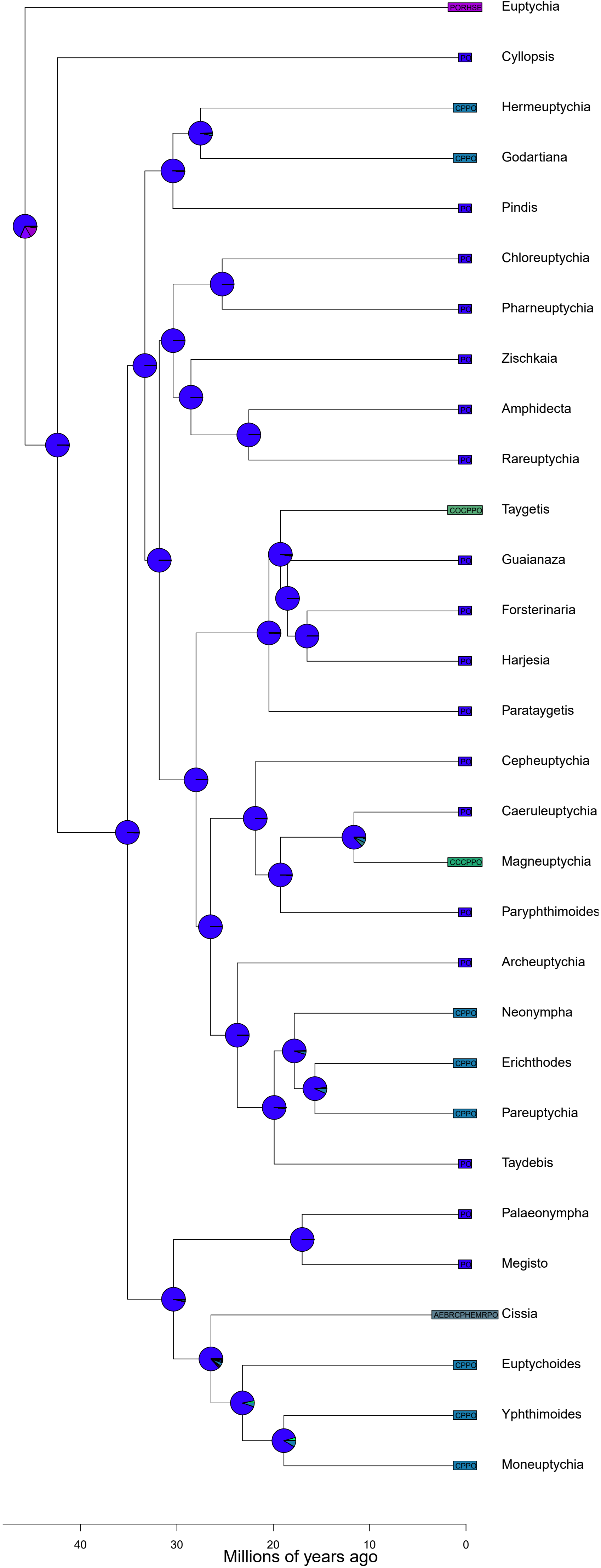

Supplement: Supplementary file 8 [file ECE3-10-3636-s009.pdf]

Family-Level Subtree 8 DEC\* Reconstruction

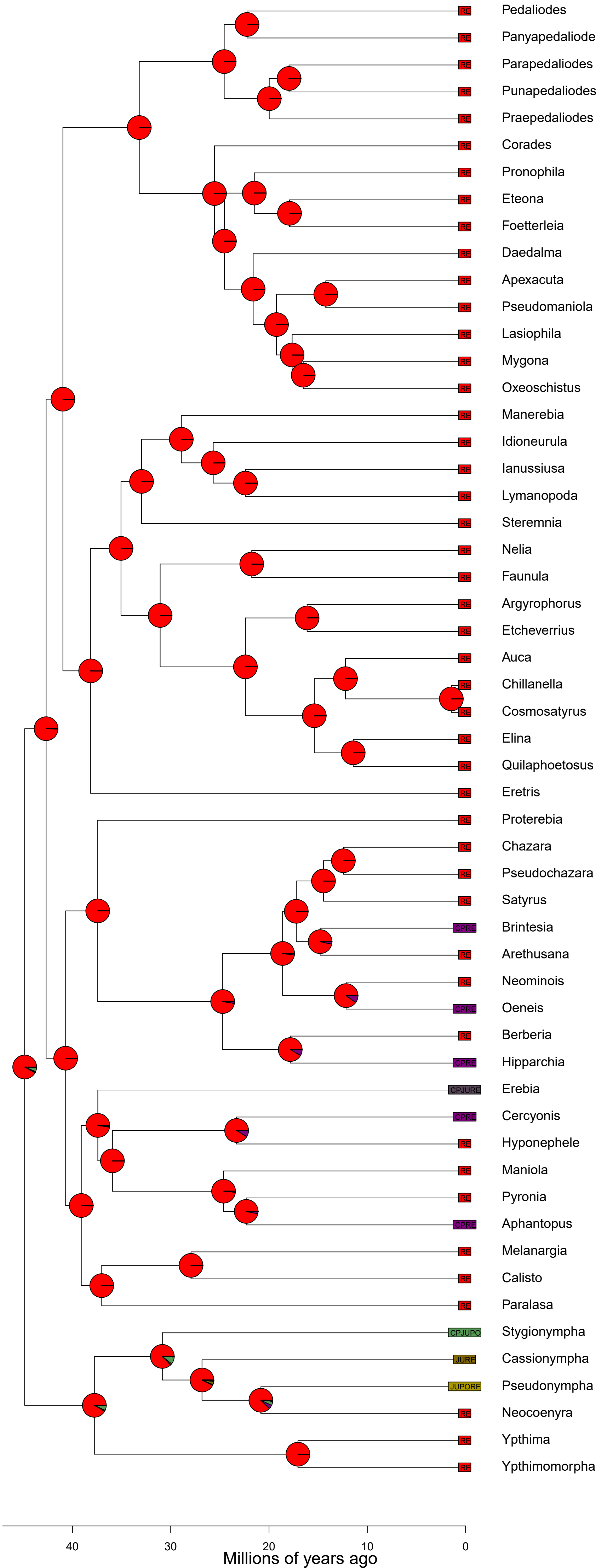

Supplement: Supplementary file 9 [file ECE3-10-3636-s010.pdf]

Family-Level Subtree 9 DEC\* Reconstruction

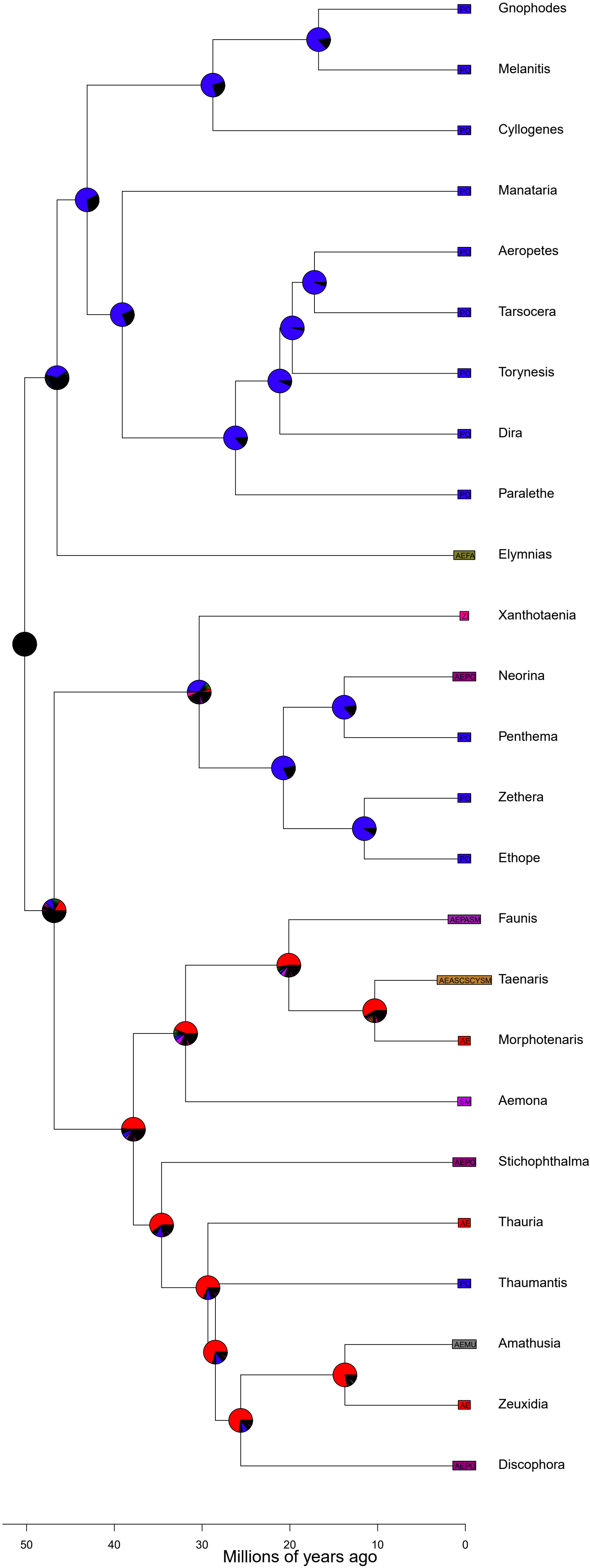

Supplement: Supplementary file 10 [file ECE3-10-3636-s011.pdf]
