# Supplementary material for: Do major host shifts spark diversification in butterflies?
Source: Ecol Evol. 2020 Feb 26;10(8):3636–46. doi: 10.1002/ece3.6116 (PMC7160180; doi:10.1002/ece3.6116)
Supplement: Supplementary file 14 [file ECE3-10-3636-s015.pdf]

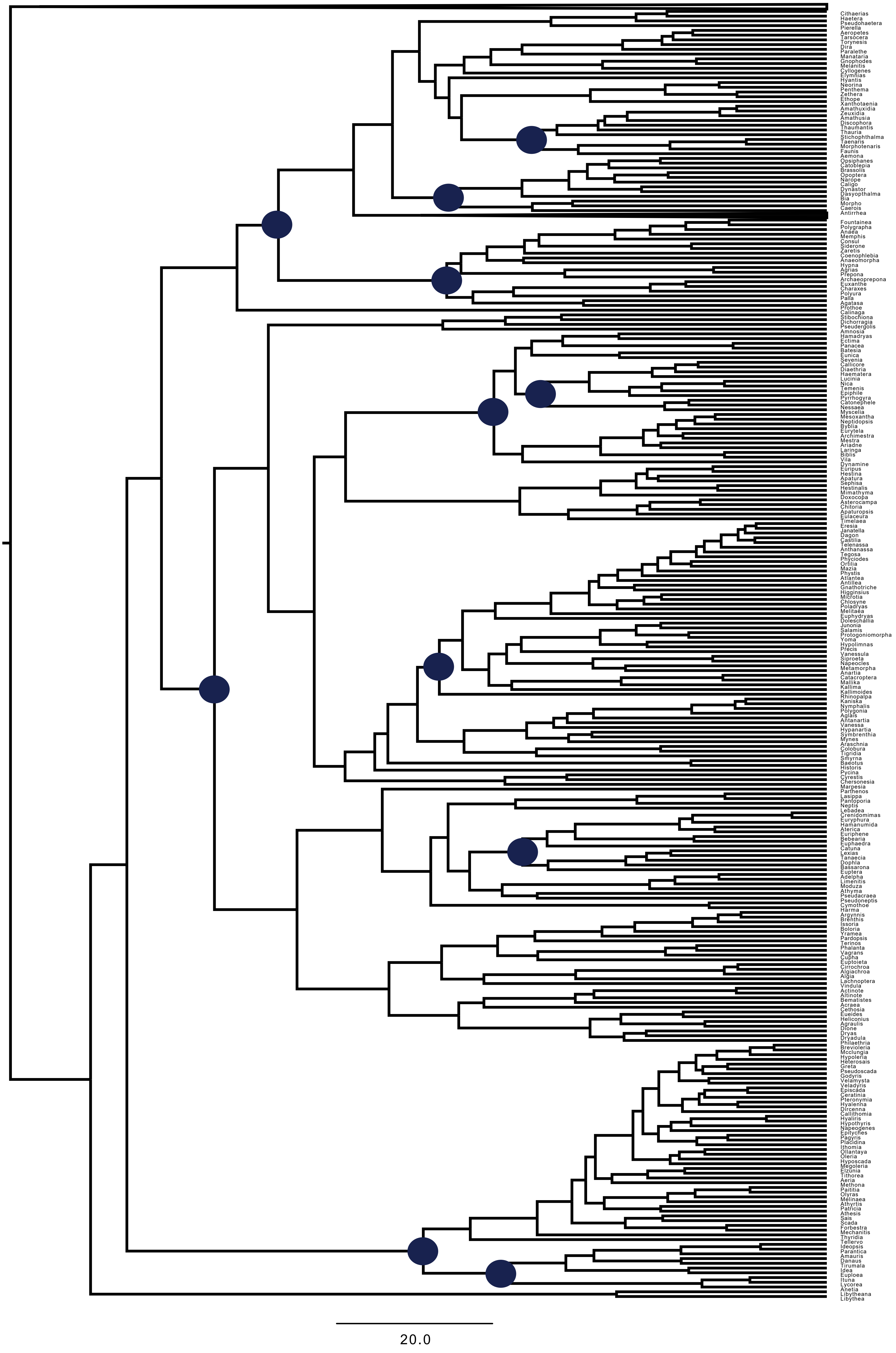
Cithaerias
Haetera
Pseudohaetera
Pierella
Aeropetes
Tarsocera
Torynesis
Dira
Paralethe
Manataria
Gnophodes
Melanitis
Cyllogenes
Elymnias
Hyantis
Neorina
Penthema
Zethera
Ethope
Xanthotaenia
Amathuxidia
Zeuxidia
Amathusia
Discophora
Thaumantis
Thauria
Stichophthalma
Taenaris
Morphotenaris
Faunis
Aemona
Opsiphanes
Catoblepia
Brassolis
Opoptera
Narope
Caligo
Dynastor
Dasyopthalma
Bia
Morpho
Caerois
Antirrhea
Fountainea
Polygrapha
Anaea
Memphis
Consul
Siderone
Zaretis
Coenophlebia
Anaeomorpha
Hypna
Agrias
Prepona
Archaeoprepona
Euxanthe
Charaxes
Polyura
Palla
Agatasa
Prothoe
Calinaga
Stibochiona
Dichorragia
Pseudergolis
Amnosia
Hamadryas
Ectima
Panacea
Batesia
Eunica
Sevenia
Callicore
Diaethria
Haematera
Lucinia
Nica
Temenis
Epiphile
Pyrrhogyra
Catonephele
Nessaea
Myscelia
Mesoxantha
Neptidopsis
Byblia
Eurytela
Archimestra
Mestra
Ariadne
Laringa
Biblis
Vila
Dynamine
Euripus
Hestina
Apatura
Sephisa
Hestinalis
Mimathyma
Doxocopa
Asterocampa
Chitoria
Apaturopsis
Eulaceura
Timelaea
Eresia
Janatella
Dagon
Castilia
Telenassa
Anthanassa
Tegosa
Phyciodes
Ortilia
Mazia
Phystis
Atlantea
Antillea
Gnathotriche
Higginsius
Microtia
Chlosyne
Poladryas
Melitaea
Euphydryas
Doleschallia
Junonia
Salamis
Protogoniomorpha
Yoma
Hypolimnas
Precis
Vanessula
Siproeta
Napeocles
Metamorpha
Anartia
Catacroptera
Mallika
Kallima
Kallimoides
Rhinopalpa
Kaniska
Nymphalis
Polygonia
Aglais
Antanartia
Vanessa
Hypanartia
Symbrenthia
Mynes
Araschnia
Colobura
Tigridia
Smyrna
Baeotus
Historis
Pycina
Cyrestis
Chersonesia
Marpesia
Parthenos
Lasippa
Pantoporia
Neptis
Lebadea
Crenidomimas
Euryphura
Hamanumida
Aterica
Euriphene
Bebearia
Euphaedra
Catuna
Lexias
Tanaecia
Dophla
Bassarona
Euptera
Adelpha
Limenitis
Moduza
Athyma
Pseudacraea
Pseudoneptis
Cymothoe
Harma
Argynnis
Brenthis
Issoria
Boloria
Yramea
Pardopsis
Terinos
Phalanta
Vagrans
Cupha
Euptoieta
Cirrochroa
Algiachroa
Algia
Lachnoptera
Vindula
Actinote
Altinote
Bematistes
Acraea
Cethosia
Eueides
Heliconius
Agraulis
Dione
Dryas
Dryadula
Philaethria
Brevioleria
Mcclungia
Hypoleria
Heterosais
Greta
Pseudoscada
Godyris
Velamysta
Veladyris
Episcada
Ceratinia
Pteronymia
Hyalenna
Dircenna
Callithomia
Hyaliris
Hypothyris
Napeogenes
Epityches
Pagyris
Placidina
Ithomia
Ollantaya
Oleria
Hyposcada
Megoleria
Elzunia
Tithorea
Aeria
Methona
Paititia
Olyras
Melinaea
Athyrtis
Patricia
Athesis
Sais
Scada
Forbestra
Mechanitis
Thyridia
Tellervo
Ideopsis
Parantica
Amauris
Danaus
Tirumala
Idea
Euploea
Ituna
Lycorea
Anetia
Libytheana
Libythea
20.0
