# Supplementary material for: Do major host shifts spark diversification in butterflies?
Source: Ecol Evol. 2020 Feb 26;10(8):3636–46. doi: 10.1002/ece3.6116 (PMC7160180; doi:10.1002/ece3.6116)
Supplement: Supplementary file 15 [file ECE3-10-3636-s002.docx]

Appendix Table S1: Nymphalid Genus-Level Waiting Time Differences as a Function of Status (Host Orders)

Residuals:

| Min | 1Q | Median | 3Q | Max |
| --- | --- | --- | --- | --- |
| -57.435 | -11.974 | 0.283 | 12.420 | 57.374 |

Coefficients:

|  | Estimate | Std. Error | t value | Pr(>\|t\|) |
| --- | --- | --- | --- | --- |
| None | -0.1141 | 1.5860 | -0.072 | 0.943 |
| Gains | -0.3356 | 7.1692 | -0.047 | 0.963 |
| Loss | -1.9161 | 6.8626 | -0.279 | 0.780 |

Residual standard error: 20.85 on 230 degrees of freedom

Multiple R-squared: 0.0003434, Adjusted R-squared: -0.008349

F-statistic: 0.03951 on 2 and 230 DF, p-value: 0.9613

Appendix Table 2: Nymphalid Genus-level Waiting Time Differences as a Function of Ecological Opportunity Indices (Host Orders)

Residuals:

| Min | 1Q | Median | 3Q | Max |
| --- | --- | --- | --- | --- |
| -26.836 | -4.861 | -1.279 | 6.023 | 32.918 |

Coefficients:

|  | Estimate | Std. Error | t value | Pr(>\|t\|) |
| --- | --- | --- | --- | --- |
| Early Adoption Index | 4.570e-01 | 3.171e-01 | 1.441 | 0.166 |
| Host Volatility | -4.693e+01 | 1.305e+02 | -0.360 | 0.723 |
| Host Age | -2.882e-01 | 2.766e-01 | -1.042 | 0.311 |
| Host Diversity | 4.748e-04 | 8.498e-04 | 0.559 | 0.583 |

---

Signif. codes: 0 ‘***’ 0.001 ‘**’ 0.01 ‘*’ 0.05 ‘.’ 0.1 ‘ ’ 1

Residual standard error: 15.86 on 19 degrees of freedom

Multiple R-squared: 0.112, Adjusted R-squared: -0.07496

F-statistic: 0.5991 on 4 and 19 DF, p-value: 0.6678

Appendix Table 3: Nymphalid Genus-level Exclusive Diversity Differences as a Function of Host-use (Host Orders)

Residuals:

| Min | 1Q | Median | 3Q | Max |
| --- | --- | --- | --- | --- |
| -2349.98 | -17.63 | 1.07 | 24.23 | 2210.95 |

Coefficients:

|  | Estimate | Std. Error | t value | Pr(>\|t\|) |
| --- | --- | --- | --- | --- |
| None | 1.633 | 10.293 | 0.159 | 0.874 |
| Gain | -17.858 | 20.978 | -0.851 | 0.395 |
| Loss | -24.651 | 39.163 | -0.629 | 0.529 |

---

Signif. codes: 0 ‘***’ 0.001 ‘**’ 0.01 ‘*’ 0.05 ‘.’ 0.1 ‘ ’ 1

Residual standard error: 238 on 804 degrees of freedom

Multiple R-squared: 0.001241, Adjusted R-squared: -0.001243

F-statistic: 0.4996 on 2 and 804 DF, p-value: 0.6069

Appendix Table 4: Nymphalid Genus-level Diversity Differences as a Function of Ecological Opportunity Indices (Host Orders)

Residuals:

| Min | 1Q | Median | 3Q | Max |
| --- | --- | --- | --- | --- |
| -1245.32 | -17.98 | 12.99 | 35.09 | 845.31 |

Coefficients:

|  | Estimate | Std. Error | t value | Pr(>\|t\|) |
| --- | --- | --- | --- | --- |
| Early Adoption Index | 1.130e+00 | 1.268e+00 | 0.891 | 0.374 |
| Host Volatility | -1.966e+02 | 2.596e+02 | -0.757 | 0.450 |
| Host Age | -9.289e-01 | 1.325e+00 | -0.701 | 0.484 |
| Host Diversity | 1.747e-04 | 1.258e-03 | 0.139 | 0.890 |

---

Signif. codes: 0 ‘***’ 0.001 ‘**’ 0.01 ‘*’ 0.05 ‘.’ 0.1 ‘ ’ 1

Residual standard error: 153.2 on 187 degrees of freedom

Multiple R-squared: 0.01613, Adjusted R-squared: -0.004911

F-statistic: 0.7667 on 4 and 187 DF, p-value: 0.5482

Appendix Table 5: Nymphalid Genus-level Inclusive Diversity Differences as a Function of Host-use Status (Host Orders)

Residuals:

| Min | 1Q | Median | 3Q | Max |
| --- | --- | --- | --- | --- |
| -2349.98 | -19.06 | -0.63 | 25.59 | 2210.95 |

Coefficients:

|  | Estimate | Std. Error | t value | Pr(>\|t\|) |
| --- | --- | --- | --- | --- |
| None | 1.633 | 10.444 | 0.156 | 0.876 |
| Gain | -23.444 | 21.286 | -1.101 | 0.271 |
| Loss | 5.168 | 39.738 | 0.130 | 0.897 |

---Signif. codes: 0 ‘***’ 0.001 ‘**’ 0.01 ‘*’ 0.05 ‘.’ 0.1 ‘ ’ 1

Residual standard error: 241.5 on 804 degrees of freedom

Multiple R-squared: 0.0016, Adjusted R-squared: -0.0008837

F-statistic: 0.6442 on 2 and 804 DF, p-value: 0.5254

Appendix Table 6: Nymphalid Genus-level Inclusive Diversity Differences as a Function of Ecological Opportunity Indices (Host Orders)

Residuals:

| Min | 1Q | Median | 3Q | Max |
| --- | --- | --- | --- | --- |
| -1302.86 | -15.31 | 20.19 | 38.05 | 885.21 |

Coefficients:

|  | Estimate | Std. Error | t value | Pr(>\|t\|) |
| --- | --- | --- | --- | --- |
| Early Adoption Index | -2.005e+00 | 1.423e+00 | -1.409 | 0.160 |
| Host Volatility | -1.178e+02 | 2.913e+02 | -0.404 | 0.686 |
| Host Age | 2.006e+00 | 1.487e+00 | 1.349 | 0.179 |
| Host Diversity | -2.478e-04 | 1.411e-03 | -0.176 | 0.861 |

---Signif. codes: 0 ‘***’ 0.001 ‘**’ 0.01 ‘*’ 0.05 ‘.’ 0.1 ‘ ’ 1

Residual standard error: 171.9 on 187 degrees of freedom

Multiple R-squared: 0.02432, Adjusted R-squared: 0.003449

F-statistic: 1.165 on 4 and 187 DF, p-value: 0.3276

Appendix Table 7: Nymphalid Genus-level Gamma Statistics as a Function of Host-use Status (Host Orders)

Iterations = 3001:999901

Thinning interval = 100

Sample size = 9970

DIC:-8.289565

G-structure: ~animal

|  | post.mean | l-95% CI | u-95% CI | eff.samp |
| --- | --- | --- | --- | --- |
| animal | 5.206 | 3.791 | 6.915 | 9970 |

R-structure: ~units

|  | post.mean | l-95% CI | u-95% CI | eff.samp |
| --- | --- | --- | --- | --- |
| units | 0.01247 | 0.001141 | 0.03441 | 9211 |

Location effects: Gamma Stat ~ Status

|  | post.mean | l-95% CI | u-95% CI | eff.samp | pMCMC |
| --- | --- | --- | --- | --- | --- |
| None | -3.9303 | -4.6992 | -3.1696 | 10544 | <1e-04 *** |
| Gain | -0.2000 | -1.4545 | 1.1361 | 9970 | 0.763 |
| Loss | -0.1756 | -1.4337 | 1.0796 | 9970 | 0.772 |

Geweke Diagnostic:

Fraction in 1st window = 0.1

Fraction in 2nd window = 0.5

| None | Gain | Loss |
| --- | --- | --- |
| -0.4081 | -0.8405 | -0.4737 |

Appendix Table 8: Nymphalid Genus-level Gamma Statistics as a Function of Ecological Opportunity Indices (Host Orders)

Iterations = 3001:999901

Thinning interval = 100

Sample size = 9970

DIC: 16.40472

G-structure: ~animal

|  | post.mean | l-95% CI | u-95% CI | eff.samp |
| --- | --- | --- | --- | --- |
| animal | 5.192 | 1.098e-12 | 14.03 | 9970 |

R-structure: ~units

|  | post.mean | l-95% CI | u-95% CI | eff.samp |
| --- | --- | --- | --- | --- |
| units | 1.597 | 0.001316 | 6.353 | 9970 |

Location effects: gamma.stat ~ Early.Bird + Host Volatility + Host Age + Host Diversity

|  | post.mean | l-95% CI | u-95% CI | eff.samp | pMCMC |
| --- | --- | --- | --- | --- | --- |
| (Intercept) | 2.816e+00 | -1.622e+01 | 2.208e+01 | 11927 | 0.7286 |
| Early Adoption Index | 2.188e-01 | 6.728e-02 | 3.704e-01 | 9970 | 0.0134 * |
| Host Volatility | 4.277e+00 | -2.433e+01 | 3.144e+01 | 9970 | 0.7157 |
| Host Age | -1.903e-01 | -4.004e-01 | 1.841e-02 | 11261 | 0.0680 . |
| Host Diversity | -4.949e-05 | -2.494e-04 | 1.267e-04 | 9970 | 0.5430 |

---

Signif. codes: 0 ‘***’ 0.001 ‘**’ 0.01 ‘*’ 0.05 ‘.’ 0.1 ‘ ’ 1

Geweke Diagnostic:

Fraction in 1st window = 0.1

Fraction in 2nd window = 0.5

| (Intercept) | Early.Bird | Host Volatility | Host Age | Host Diversity |
| --- | --- | --- | --- | --- |
| 1.8328 | 0.7729 | -1.3619 | -1.7799 | 0.3771 |

Appendix Table 9: Nymphalid Genus-Level Waiting Time Differences as a Function of Status (Host Families)

Residuals:

| Min | 1Q | Median | 3Q | Max |
| --- | --- | --- | --- | --- |
| -55.505 | -11.884 | 0.608 | 12.991 | 46.967 |

Coefficients:

|  | Estimate | Std. Error | t value | Pr(>\|t\|) |
| --- | --- | --- | --- | --- |
| None | -0.6385 | 1.7522 | -0.364 | 0.716 |
| Gains | 10.3272 | 17.5839 | 0.587 | 0.558 |
| Loss | 3.7037 | 13.1261 | 0.282 | 0.778 |

Residual standard error: 18.18 on 131 degrees of freedom

Multiple R-squared: 0.003197, Adjusted R-squared: -0.01202

F-statistic: 0.2101 on 2 and 131 DF, p-value: 0.8108

Appendix Table 10: Nymphalid Genus-level Waiting Time Difference as a Function of Ecological Opportunity Indices (Host Families)

Residuals:

ALL 4 residuals are 0: no residual degrees of freedom!

Coefficients:

|  | Estimate | Std. Error | t value | Pr(>\|t\|) |
| --- | --- | --- | --- | --- |
| Early Adoption Index | 0.43170 | NA | NA | NA |
| Host Volatility | -1.71051 | NA | NA | NA |
| Host Age | -0.44402 | NA | NA | NA |
| Host Diversity | 0.00487 | NA | NA | NA |

---Signif. codes: 0 ‘***’ 0.001 ‘**’ 0.01 ‘*’ 0.05 ‘.’ 0.1 ‘ ’ 1

Residual standard error: NaN on 0 degrees of freedom

Multiple R-squared: 1, Adjusted R-squared: NaN

F-statistic: NaN on 4 and 0 DF, p-value: NA

Appendix Table 11: Nymphalid Genus-level Exclusive Diversity Differences as a Function of Host-use Status (Host Families)

Residuals:

| Min | 1Q | Median | 3Q | Max |
| --- | --- | --- | --- | --- |
| -638.13 | -13.13 | -0.13 | 19.30 | 631.87 |

Coefficients:

|  | Estimate | Std. Error | t value | Pr(>\|t\|) |
| --- | --- | --- | --- | --- |
| None | 3.347 | 4.229 | 0.791 | 0.4291 |
| Gain | -16.365 | 8.019 | -2.041 | 0.0418 * |
| Loss | -36.938 | 17.034 | -2.169 | 0.0306 * |

---Signif. codes: 0 ‘***’ 0.001 ‘**’ 0.01 ‘*’ 0.05 ‘.’ 0.1 ‘ ’ 1

Residual standard error: 78.14 on 528 degrees of freedom

Multiple R-squared: 0.01464, Adjusted R-squared: 0.0109

F-statistic: 3.922 on 2 and 528 DF, p-value: 0.02039

Appendix Table 12: Nymphalid Genus-level Exclusive Diversity Differences as a Function of Ecological Opportunity Indices (Host Families)

Residuals:

| Min | 1Q | Median | 3Q | Max |
| --- | --- | --- | --- | --- |
| -321.56 | -5.20 | 5.05 | 19.44 | 100.50 |

Coefficients:

|  | Estimate | Std. Error | t value | Pr(>\|t\|) |
| --- | --- | --- | --- | --- |
| Early Adoption Index | -0.4555273 | 1.6073393 | -0.283 | 0.777 |
| Host Volatility | 50.8440592 | 38.1510208 | 1.333 | 0.185 |
| Host Age | 0.3806447 | 1.6048426 | 0.237 | 0.813 |
| Host Diversity | -0.0007372 | 0.0007305 | -1.009 | 0.315 |

---

Signif. codes: 0 ‘***’ 0.001 ‘**’ 0.01 ‘*’ 0.05 ‘.’ 0.1 ‘ ’ 1

Residual standard error: 55.55 on 131 degrees of freedom

Multiple R-squared: 0.08809, Adjusted R-squared: 0.06024

F-statistic: 3.164 on 4 and 131 DF, p-value: 0.01612

Appendix Table 13: Nymphalid Genus-level Inclusive Diversity Differences as a Function of Host-use Status (Host Families)

Residuals:

| Min | 1Q | Median | 3Q | Max |
| --- | --- | --- | --- | --- |
| -611.14 | -12.35 | -1.01 | 19.15 | 630.46 |

Coefficients:

|  | Estimate | Std. Error | t value | Pr(>\|t\|) |
| --- | --- | --- | --- | --- |
| None | 3.347 | 4.242 | 0.789 | 0.4304 |
| Gain | -20.497 | 8.043 | -2.549 | 0.0111 * |
| Loss | -35.878 | 17.085 | -2.100 | 0.0362 * |

---Signif. codes: 0 ‘***’ 0.001 ‘**’ 0.01 ‘*’ 0.05 ‘.’ 0.1 ‘ ’ 1

Residual standard error: 78.37 on 528 degrees of freedom

Multiple R-squared: 0.01798, Adjusted R-squared: 0.01426

F-statistic: 4.835 on 2 and 528 DF, p-value: 0.008306

Appendix Table 14: Nymphalid Genus-level Inclusive Diversity Differences as a Function of Ecological Opportunity Indices (Host Families)

Residuals:

| Min | 1Q | Median | 3Q | Max |
| --- | --- | --- | --- | --- |
| -315.101 | -10.199 | 6.786 | 21.615 | 104.233 |

Coefficients:

|  | Estimate | Std. Error | t value | Pr(>\|t\|) |
| --- | --- | --- | --- | --- |
| Early Adoption Index | -0.2979736 | 1.6486391 | -0.181 | 0.8569 |
| Host Volatility | 32.4692473 | 39.1312915 | 0.830 | 0.4082 |
| Host Age | 0.2257809 | 1.6460782 | 0.137 | 0.8911 |
| Host Diversity | -0.0012417 | 0.0007493 | -1.657 | 0.0999 . |

---Signif. codes: 0 ‘***’ 0.001 ‘**’ 0.01 ‘*’ 0.05 ‘.’ 0.1 ‘ ’ 1

Residual standard error: 56.98 on 131 degrees of freedom

Multiple R-squared: 0.1107, Adjusted R-squared: 0.08357

F-statistic: 4.078 on 4 and 131 DF, p-value: 0.003789

Appendix Table 15: Nymphalid Genus-level Gamma Statistics as a Function of Host-use Status (Host Families)

Iterations = 3001:999901

Thinning interval = 100

Sample size = 9970

DIC: 9.296147

G-structure: ~animal

|  | post.mean | l-95% CI | u-95% CI | eff.samp |
| --- | --- | --- | --- | --- |
| animal | 0.4068 | 1.889e-07 | 1.279 | 7243 |

R-structure: ~units

|  | post.mean | l-95% CI | u-95% CI | eff.samp |
| --- | --- | --- | --- | --- |
| units | 0.132 | 0.001445 | 0.5009 | 9495 |

Location effects: Gamma Stat ~ Status

|  | post.mean | l-95% CI | u-95% CI | eff.samp | pMCMC |
| --- | --- | --- | --- | --- | --- |
| (Intercept) | -3.7639 | -4.3648 | -3.2051 | 9970 | <1e-04 *** |
| Gain | 0.9105 | -1.5147 | 3.3517 | 9977 | 0.449 |
| Loss | 0.8769 | -0.9921 | 2.8689 | 10301 | 0.362 |

Geweke Diagnostic:

Fraction in 1st window = 0.1

Fraction in 2nd window = 0.5

| None | Gain | Loss |
| --- | --- | --- |
| 0.06493 | -0.02890 | 0.41701 |

Appendix Table 16: Nymphalid Genus-level Gamma Statistics as a Function of Ecological Opportunity Indices (Host Families)

Iterations = 3001:999901

Thinning interval = 100

Sample size = 9970

DIC: 15.62889

G-structure: ~animal

|  | post.mean | l-95% CI | u-95% CI | eff.samp |
| --- | --- | --- | --- | --- |
| animal |  |  |  |  |

R-structure: ~units

|  | post.mean | l-95% CI | u-95% CI | eff.samp |
| --- | --- | --- | --- | --- |
| units |  |  |  |  |

Location effects: X0 ~ Early.Bird

|  | post.mean | l-95% CI | u-95% CI | eff.samp | pMCMC |
| --- | --- | --- | --- | --- | --- |
| (Intercept) | NA | NA | NA | NA | NA |
| Early Adoption Index | NA | NA | NA | NA | NA |
| Host Volatility | NA | NA | NA | NA | NA |
| Host Age | NA | NA | NA | NA | NA |
| Host Diversity | NA | NA | NA | NA | NA |

---

Signif. codes: 0 ‘***’ 0.001 ‘**’ 0.01 ‘*’ 0.05 ‘.’ 0.1 ‘ ’ 1

Appendix Table 17: Nymphalid Species-level Waiting Time Differences as a Function of Host-use Status (Host Families)

Residuals:

| Min | 1Q | Median | 3Q | Max |
| --- | --- | --- | --- | --- |
| -63.282 | -6.235 | 0.170 | 5.857 | 49.354 |

Coefficients:

|  | Estimate | Std. Error | t value | Pr(>\|t\|) |
| --- | --- | --- | --- | --- |
| None | 0.4838 | 1.1537 | 0.675 | 0.675 |
| Gains | -6.8354 | 4.4507 | -1.536 | 0.126 |
| Loss | 0.4084 | 5.6749 | 0.072 | 0.943 |

Residual standard error: 13.87 on 262 degrees of freedom

Multiple R-squared: 0.00901, Adjusted R-squared: 0.001446

F-statistic: 1.191 on 2 and 262 DF, p-value: 0.3055

Appendix Table 18: Nymphalid Species-level Waiting Time Differences as a Function of Ecological Opportunity Indices (Host Families)

Residuals:

| Min | 1Q | Median | 3Q | Max |
| --- | --- | --- | --- | --- |
| -34.350 | -4.446 | 1.374 | 3.075 | 14.255 |

Coefficients:

|  | Estimate | Std. Error | t value | Pr(>\|t\|) |
| --- | --- | --- | --- | --- |
| Early Adoption Index | -0.5222174 | 0.3950076 | -1.322 | 0.196 |
| Host Volatility | 8.9526671 | 28.8686880 | 0.310 | 0.758 |
| Host Age | 0.4168549 | 0.3703071 | 1.126 | 0.269 |
| Host Diversity | -0.0006775 | 0.0011938 | -0.567 | 0.574 |

---

Signif. codes: 0 ‘***’ 0.001 ‘**’ 0.01 ‘*’ 0.05 ‘.’ 0.1 ‘ ’ 1

Residual standard error: 11.99 on 32 degrees of freedom

(2 observations deleted due to missingness)

Multiple R-squared: 0.1054, Adjusted R-squared: -0.006477

F-statistic: 0.9421 on 4 and 32 DF, p-value: 0.4523

Appendix Table 19: Nymphalid Species-level Inclusive Diversity Differences as a Function of Host -use Status (Host Families)

| Min | 1Q | Median | 3Q | Max |
| --- | --- | --- | --- | --- |
| -457.51 | -8.49 | -3.60 | 1.29 | 607.19 |

Coefficients:

|  | Estimate | Std. Error | t value | Pr(>\|t\|) |
| --- | --- | --- | --- | --- |
| None | 8.488 | 2.303 | 3.686 | 0.00024 *** |
| Gain | -9.782 | 4.107 | -2.382 | 0.01740 * |
| Loss | -5.379 | 6.946 | 0.43889 | 0.43889 |

---Signif. codes: 0 ‘***’ 0.001 ‘**’ 0.01 ‘*’ 0.05 ‘.’ 0.1 ‘ ’ 1

Residual standard error: 52.66 on 991 degrees of freedom

Multiple R-squared: 0.005807, Adjusted R-squared: 0.0038

F-statistic: 2.894 on 2 and 991 DF, p-value: 0.05582

Appendix Table 20: Nymphalid Species-level Inclusive Diversity Differences as a Function of Ecological Opportunity Indices (Host Families)

Residuals:

| Min | 1Q | Median | 3Q | Max |
| --- | --- | --- | --- | --- |
| -23.1200 | -0.8374 | 0.8360 | 1.3812 | 26.1504 |

Coefficients:

|  | Estimate | Std. Error | t value | Pr(>\|t\|) |
| --- | --- | --- | --- | --- |
| Early Adoption Index | -0.5680069 | 0.0867218 | -6.550 | 3.16e-10 *** |
| Host Volatility | -0.9892847 | 1.6080669 | -0.615 | 0.539 |
| Host Age | 0.5555549 | 0.0862351 | 6.442 | 5.83e-10 *** |
| Host Diversity | -0.0001471 | 0.0001148 | -1.281 | 0.201 |

---Signif. codes: 0 ‘***’ 0.001 ‘**’ 0.01 ‘*’ 0.05 ‘.’ 0.1 ‘ ’ 1

Residual standard error: 4.309 on 255 degrees of freedom

(20 observations deleted due to missingness)

Multiple R-squared: 0.186, Adjusted R-squared: 0.1733

F-statistic: 14.57 on 4 and 255 DF, p-value: 9.888e-11

Appendix Table 21: Nymphalid Species-level Gamma Statistics as a Function of Host-use Status (Host Families)

Iterations = 3001:999901

Thinning interval = 100

Sample size = 9970

DIC:- -381.6454

G-structure: ~animal

|  | post.mean | l-95% CI | u-95% CI | eff.samp |
| --- | --- | --- | --- | --- |
| animal | 2.422 | 1.942 | 2.978 | 7883 |

R-structure: ~units

|  | post.mean | l-95% CI | u-95% CI | eff.samp |
| --- | --- | --- | --- | --- |
| units | 0.003789 | 0.0007557 | 0.008757 | 9489 |

Location effects: Gamma Stat ~ Status

|  | post.mean | l-95% CI | u-95% CI | eff.samp | pMCMC |
| --- | --- | --- | --- | --- | --- |
| None | -1.30579 | -1.62737 | -0.95455 | 9970 | <1e-04 *** |
| Gain | -0.08588 | -0.86546 | 0.72151 | 9970 | 0.833 |
| Loss | -0.08572 | -0.90135 | 0.68354 | 9970 | 0.829 |

Appendix Table 22: Nymphalid Species-level Gamma Statistics as a Function of Ecological Opportunity Indices (Host Families)

Iterations = 3001:999901

Thinning interval = 100

Sample size = 9970

DIC: 14.6317

G-structure: ~animal

|  | post.mean | l-95% CI | u-95% CI | eff.samp |
| --- | --- | --- | --- | --- |
| animal | 8.835 | 1.097e-06 | 25.62 | 5540 |

R-structure: ~units

|  | post.mean | l-95% CI | u-95% CI | eff.samp |
| --- | --- | --- | --- | --- |
| units | 3.258 | 0.002017 | 12.5 | 9688 |

Location effects: gamma.stat ~ Early.Bird + Host Volatility + Host Age + Host Diversity

|  | post.mean | l-95% CI | u-95% CI | eff.samp | pMCMC |
| --- | --- | --- | --- | --- | --- |
| (Intercept) | -0.985787 | -10.325116 | 8.668767 | 9970 | 0.794 |
| Early Adoption Index | -0.043505 | -0.212019 | 0.132383 | 9613 | 0.531 |
| Host Volatility | -1.120874 | -17.971765 | 15.199878 | 9970 | 0.857 |
| Host Age | 0.042517 | -0.127680 | 0.215775 | 9970 | 0.542 |
| Host Diversity | -0.002255 | -0.007941 | 0.003002 | 9663 | 0.321 |

---

Signif. codes: 0 ‘***’ 0.001 ‘**’ 0.01 ‘*’ 0.05 ‘.’ 0.1 ‘ ’ 1

Appendix Table S23: Geiger Likelihood Models

| Host | Early Burst Likelihood | Null Likelihood | P-Value |
| --- | --- | --- | --- |
| Acanthaceae | -24.2705889166749 | -24.4756559805568 | 1 |
| Achariaceae | -6.80384015822068 | -6.82545068855855 | 1 |
| Amaranthaceae | -30.8217134313346 | -33.4444478391127 | 1 |
| Anacardiaceae | -34.8693627693718 | -35.7662127144548 | 1 |
| Annonaceae | -47.6983901313256 | -47.7073509146823 | 1 |
| Apiaceae | -262.702781432219 | -6.79364646956203 | 2.55E-113 |
| Apocynaceae | -83.970976114268 | -62.2157567766798 | 4.22E-11 |
| Aquifoliaceae | -18.701618428438 | -18.8102560659757 | 1 |
| Araceae | -14.5980476059492 | -14.603123091087 | 1 |
| Araliaceae | -6.55692632510564 | -6.57810108152929 | 1 |
| Araucariaceae | -6.93094951394479 | -7.30260109705276 | 1 |
| Arecaceae | -69.7115690138446 | -69.769321530938 | 1 |
| Aristolochiaceae | -12.4817130751343 | -13.4698431237797 | 1 |
| Asparagaceae | -22.1808653509207 | -23.6966448728518 | 1 |
| Asteraceae | -67.4035878782108 | -67.773112229075 | 1 |
| Berberidaceae | -6.92220757967273 | -7.17499856502485 | 1 |
| Betulaceae | -26.6029339190823 | -29.3232600080215 | 1 |
| Bignoniaceae | -34.8711261705137 | -36.3031774972318 | 1 |
| Boraginaceae | -262.702781432218 | -26.2210235137557 | 7.26E-105 |
| Brassicaceae | -17.5022156418202 | -19.4169952117311 | 1 |
| Bromeliaceae | -17.4019664014372 | -17.8436178199958 | 1 |
| Burseraceae | -12.3048329912621 | -12.3979018524393 | 1 |
| Calophyllaceae | -6.89279064975089 | -7.01725583377264 | 1 |
| Cannabaceae | -28.470004563345 | -28.9418629270413 | 1 |
| Cannaceae | -6.88593921398943 | -6.99489573452801 | 1 |
| Capparaceae | -6.68367580108751 | -6.68393692895186 | 1 |
| Caprifoliaceae | -36.2472205256356 | -36.7901343455852 | 1 |
| Caricaceae | -6.93562612174448 | -8.00939866131714 | 1 |
| Caryocaraceae | -6.89279062023153 | -7.01725583377264 | 1 |
| Caryophyllaceae | -6.93094951396764 | -7.30260109705276 | 1 |
| Casuarinaceae | -6.91800013899589 | -7.14056717170542 | 1 |
| Celastraceae | -24.5534575377609 | -24.9445791389883 | 1 |
| Chrysobalanaceae | -9.52930722550898 | -11.6875200443071 | 1 |
| Clethraceae | -1.38629462213216 | -5.73203587136837 | 1 |
| Clusiaceae | -34.8619350614944 | -35.4572025497014 | 1 |
| Combretaceae | -26.6052869233509 | -26.8822891699412 | 1 |
| Commelinaceae | -22.1808363360853 | -22.1872694951667 | 1 |
| Connaraceae | -12.3901457461315 | -12.5726751287719 | 1 |
| Convolvulaceae | -42.5129360296963 | -43.8613258671962 | 1 |
| Costaceae | -12.4820355390037 | -13.568766898775 | 1 |
| Crassulaceae | -12.3497384391058 | -12.4572566104912 | 1 |
| Cucurbitaceae | -11.5179450989476 | -11.6605185066158 | 1 |
| Cycadaceae | -12.482035595545 | -13.8107082549629 | 1 |
| Cyclanthaceae | -12.4817073678076 | -13.4505691420422 | 1 |
| Cyperaceae | -262.702781432218 | -102.88318605514 | 1.74E-71 |
| Dichapetalaceae | -12.479699791311 | -13.0033091805488 | 1 |
| Dilleniaceae | -12.4810190894205 | -13.7589457466328 | 1 |
| Dioscoreaceae | -6.9355719499748 | -7.95786493250361 | 1 |
| Dipterocarpaceae | -6.93492591056769 | -7.58170471853563 | 1 |
| Ebenaceae | -6.92025085903114 | -7.15806434882404 | 1 |
| Elaeocarpaceae | -6.89279062324096 | -7.01725583377264 | 1 |
| Ericaceae | -22.1804343354287 | -24.9629373096855 | 1 |
| Erythroxylaceae | -16.2128019005946 | -16.8608974602591 | 1 |
| Euphorbiaceae | -262.702781432219 | -100.344058386746 | 1.36E-72 |
| Fabaceae | -98.0211968133319 | -98.639803044315 | 1 |
| Fagaceae | -30.821329372525 | -33.8409568304083 | 1 |
| Garryaceae | -6.92773033332384 | -7.23998943410325 | 1 |
| Geraniaceae | -12.1261475637004 | -12.1375813278419 | 1 |
| Gesneriaceae | -17.4891850144841 | -18.5532704674518 | 1 |
| Grossulariaceae | -14.2437622150418 | -14.3289923091088 | 1 |
| Heliconiaceae | -26.3281409559336 | -26.7255633018473 | 1 |
| Hernandiaceae | -6.92252612161765 | -7.17815932133376 | 1 |
| Humiriaceae | -6.93504307531246 | -7.62137303026753 | 1 |
| Hypoxidaceae | -6.9307425281913 | -7.29691749509513 | 1 |
| Icacinaceae | -12.4808515256176 | -13.3863085518521 | 1 |
| Iridaceae | -12.4780533138332 | -13.413011272161 | 1 |
| Juglandaceae | -6.77360595501313 | -6.78344785339499 | 1 |
| Juncaceae | -12.2509321218185 | -12.2985775963058 | 1 |
| Lacistemataceae | -6.77360595495654 | -6.78344785339499 | 1 |
| Lamiaceae | -26.5984523132069 | -28.6104079522117 | 1 |
| Laminaceae | -6.73085202521106 | -6.73252402695871 | 1 |
| Lauraceae | -46.2156951340432 | -45.2558838088449 | 0.165898265096917 |
| Lecythidaceae | -6.93094951431532 | -7.30260109705276 | 1 |
| Liliaceae | -12.4822664176156 | -14.57806183266 | 1 |
| Linaceae | -6.68561363052249 | -6.68581105918832 | 1 |
| Loranthaceae | -6.93441109400682 | -7.48380570737111 | 1 |
| Lythraceae | -6.9226115726712 | -7.17874402988691 | 1 |
| Malpighiaceae | -38.7795977926754 | -39.6588721969522 | 1 |
| Malvaceae | -89.328849701096 | -90.9415349019118 | 1 |
| Marantaceae | -30.6597451388631 | -31.3198987317011 | 1 |
| Melastomataceae | -17.5001219468834 | -18.9093702233436 | 1 |
| Meliaceae | -27.0320957584463 | -27.3827137993738 | 1 |
| Melianthaceae | -12.4605719076862 | -12.7333980040201 | 1 |
| Menispermaceae | -21.5618574001633 | -21.5621194326342 | 1 |
| Monimiaceae | -22.1638369349807 | -22.6806337340504 | 1 |
| Moraceae | -62.6909090617541 | -62.7145784949122 | 1 |
| Musaceae | -33.602661890997 | -33.7152352340827 | 1 |
| Myrtaceae | -22.1757515880637 | -22.5066367462364 | 1 |
| Neckeraceae | -6.39971589230197 | -6.47572158989062 | 1 |
| Nyctaginaceae | -9.06259944595364 | -11.0294199840162 | 1 |
| Ochnaceae | -30.8165730133795 | -32.6931880681823 | 1 |
| Oleaceae | -26.6027905407189 | -27.5705513020729 | 1 |
| Onagraceae | -12.4608341526092 | -13.0113438923752 | 1 |
| Orchidaceae | -6.3702240818603 | -6.45898136037853 | 1 |
| Pandanaceae | -12.4769129863842 | -13.3314677502581 | 1 |
| Papaveraceae | -6.78125808774484 | -6.79364646956203 | 1 |
| Passifloraceae | -36.464501881233 | -36.7912871843928 | 1 |
| Phrymaceae | -6.92057344658009 | -7.16069446736797 | 1 |
| Phyllanthaceae | -1.38629462213216 | -5.73203587136837 | 1 |
| Piperaceae | -38.7740735707876 | -40.0392505528978 | 1 |
| Plantaginaceae | -38.6792479280904 | -39.7981969479569 | 1 |
| Poaceae | -104.282067331842 | -104.363319882621 | 1 |
| Polygonaceae | -26.6028081446915 | -26.8777721274971 | 1 |
| Polypodiaceae | -6.93220977514693 | -7.3407239669079 | 1 |
| Portulacaceae | -12.3497384670579 | -12.4572566104912 | 1 |
| Primulaceae | -30.8213877036606 | -34.1548439292658 | 1 |
| Proteaceae | -10.9508138204275 | -12.0147237317404 | 1 |
| Pteridaceae | -6.89436932227414 | -7.0230947160647 | 1 |
| Ranunculaceae | -1.38629462213216 | -5.73203587136837 | 1 |
| Restionaceae | -12.4353504828232 | -12.7419008744681 | 1 |
| Rhamnaceae | -56.6038842251682 | -60.0545419503126 | 1 |
| Rhizophoraceae | -6.92261157268639 | -7.17874402988691 | 1 |
| Rosaceae | -63.1341643099507 | -55.0112997048474 | 5.56E-05 |
| Rubiaceae | -50.7551051791666 | -50.7583654413512 | 1 |
| Rutaceae | -34.6714507795428 | -35.2679226063573 | 1 |
| Sabiaceae | -12.3863091782338 | -12.5597554301038 | 1 |
| Salicaceae | -76.1727885026932 | -76.510966964234 | 1 |
| Sapindaceae | -61.1099744227157 | -61.2457360890633 | 1 |
| Sapotaceae | -42.5223430421839 | -44.345028652158 | 1 |
| Saxifragaceae | -6.93414539721374 | -7.45278698042378 | 1 |
| Scrophulariaceae | -49.3739645740233 | -50.2247274977083 | 1 |
| Selaginellaceae | -11.4515469247845 | -11.5874016927412 | 1 |
| Simaroubaceae | -1.38629462213217 | -5.73203587136837 | 1 |
| Smilacaceae | -25.956329866151 | -26.2951885632985 | 1 |
| Solanaceae | -42.6087401725715 | -43.1376521891618 | 1 |
| Surianaceae | -6.92261157391639 | -7.17874402988691 | 1 |
| Theaceae | -6.90534845499983 | -7.06777937358849 | 1 |
| Typhaceae | -12.4820337799824 | -14.2031982571009 | 1 |
| Ulmaceae | -73.0465754509317 | -73.1401209199552 | 1 |
| Urticaceae | -88.0350410908002 | -88.0555673055626 | 1 |
| Verbenaceae | -262.702781432205 | -78.0242702596041 | 2.58E-82 |
| Violaceae | -35.4508362741492 | -35.6237792430673 | 1 |
| Vitaceae | -1.38629462213216 | -5.73203587136837 | 1 |
| Vochysiaceae | -6.93516147948022 | -7.67143530421434 | 1 |
| Xyridaceae | -6.9342605667635 | -7.46447514772497 | 1 |
| Zingiberaceae | -30.6253427877809 | -31.3765646160151 | 1 |

The log-likelihood of early-burst fitted model and a non-transformed model (both equal-rates models) for each host family (as fitted in geiger) and the p-value of the log likelihood ration comparing the two models. A p-value of <0.05 indicates the early-burst model is significantly better than the null, non-transformed model.
